# Trapping the HIV-1 V3 loop in a helical conformation enables broad neutralization

**DOI:** 10.1038/s41594-023-01062-z

**Published:** 2023-08-21

**Authors:** Matthias Glögl, Nikolas Friedrich, Gabriele Cerutti, Thomas Lemmin, Young D. Kwon, Jason Gorman, Liridona Maliqi, Peer R. E. Mittl, Maria C. Hesselman, Daniel Schmidt, Jacqueline Weber, Caio Foulkes, Adam S. Dingens, Tatsiana Bylund, Adam S. Olia, Raffaello Verardi, Thomas Reinberg, Nicolas S. Baumann, Peter Rusert, Birgit Dreier, Lawrence Shapiro, Peter D. Kwong, Andreas Plückthun, Alexandra Trkola

**Affiliations:** 1 Institute for Medical Virology, University of Zurich (UZH), Zurich, Switzerland; 2 Zuckerman Mind Brain Behavior Institute, Columbia University, New York, NY USA; 3 Vaccine Research Center, National Institute of Allergy and Infectious Diseases, National Institutes of Health, Bethesda, MD USA; 4 Department of Biochemistry, University of Zurich (UZH), Zurich, Switzerland; 5 Fred Hutchinson Cancer Research Center, Seattle, WA USA; 6 Department of Biochemistry and Molecular Biophysics, Columbia University, New York, NY USA

**Keywords:** Virology, Immunology, Structural biology, Proteins

## Abstract

The third variable (V3) loop on the human immunodeficiency virus 1 (HIV-1) envelope glycoprotein trimer is indispensable for virus cell entry. Conformational masking of V3 within the trimer allows efficient neutralization via V3 only by rare, broadly neutralizing glycan-dependent antibodies targeting the closed prefusion trimer but not by abundant antibodies that access the V3 crown on open trimers after CD4 attachment. Here, we report on a distinct category of V3-specific inhibitors based on designed ankyrin repeat protein (DARPin) technology that reinstitute the CD4-bound state as a key neutralization target with up to >90% breadth. Broadly neutralizing DARPins (bnDs) bound V3 solely on open envelope and recognized a four-turn amphipathic α-helix in the carboxy-terminal half of V3 (amino acids 314–324), which we termed ‘αV3C’. The bnD contact surface on αV3C was as conserved as the CD4 binding site. Molecular dynamics and escape mutation analyses underscored the functional relevance of αV3C, highlighting the potential of αV3C-based inhibitors and, more generally, of postattachment inhibition of HIV-1.

## Abstract (web summary)

The third variable (V3) loop on the HIV-1 Env glycoprotein is required for viral entry. Here, the authors applied DARPin technology to produce broadly neutralizing inhibitors targeting a region of V3 that becomes accessible after binding to the CD4 receptor.

## Main

Efficient shielding of the V3 loop on envelope glycoprotein gp120 within the HIV-1 envelope (Env) trimer is paramount to protect its key function in coreceptor binding against neutralizing antibodies^1–4^. The closed Env trimer conformation severely restricts access to the V3. Only rare broadly neutralizing antibodies (bnAbs) are directed to V3, recognizing its GDIR motif and surrounding glycans and achieving up to 80% neutralization breadth^5–7^. Upon CD4 receptor engagement, the Env trimer relaxes the conformational shielding to expose the coreceptor binding site including the protruding flexible V3 for interaction with either C-C chemokine receptor type 5 (CCR5) or C-X-C chemokine receptor type 4 (CXCR4) coreceptors. The potential for neutralization of these open Env intermediates by gp120-specific antibodies has been perceived as limited due to spatial and time constraints on antibody access to the relevant epitopes^8,9^. The abundant, non-neutralizing or weakly neutralizing V3 crown antibodies, which do not interact with glycans, typically display high neutralization potency against strains with pronounced open Env states^1,10–14^. However, no means have been found to exploit the highly immunogenic V3 crown for HIV-1 prevention. On the contrary, to allow relevant neutralizing antibody responses to occur, it is considered critical to occlude the immunodominant V3 on immunogens to prevent non-neutralizing V3 crown antibodies from dictating the immune response^15–19^.

However, accumulating evidence suggests that in specific cases relevant neutralization via the V3 loop may nevertheless occur. We recently created open Env targeting V3 crown inhibitors based on the DARPin scaffold that display extraordinary breadth matching V3 glycan-targeting bnAbs^20^. Similarly, some V3 crown-specific antibodies exhibiting preference for open Env show notable breadth^20,21^. These observations suggest that neutralization of open Env after CD4 engagement may have been underestimated, and information on transitioning Env structure intermediates following CD4 receptor engagement will be critical to potentially exploit this stage for broad neutralization. However, structure definition of intermediate entry steps is limited, as trimer opening generates highly flexible regions that cannot be resolved by conventional structure analysis, including cryogenic electron microscopy (cryo-EM)^22,23^.

Here, we hypothesized that targeting distinct conformations of the V3 beyond the V3 crown may overcome restrictions of V3 for neutralization, enabling broad and potent inhibition. We used conformation-sensitive DARPins in combination with structural analysis^20^ to overcome restrictions in resolving V3 flexible structure elements. DARPins are small binding proteins (14–18 kDa) designed based on a consensus derived from naturally occurring ankyrin repeats^24–26^. Their pronounced conformation-dependent mode of target binding renders them attractive tools for stabilizing distinct epitope configurations^27^. To elucidate neutralization-relevant V3 conformations, we selected an array of bnDs that form a class of inhibitors interacting with the V3 stem, which is solely accessible on open, CD4-triggered Env. Structural analysis identified a previously unrecognized α-helix in the C-terminal V3 strand (designated αV3C) bound by these bnDs, highlighting the potential of entry process intermediates as targets for broad neutralization.

## Results

### A category of V3 stem-dependent broadly neutralizing inhibitors

**Fig. 1:**
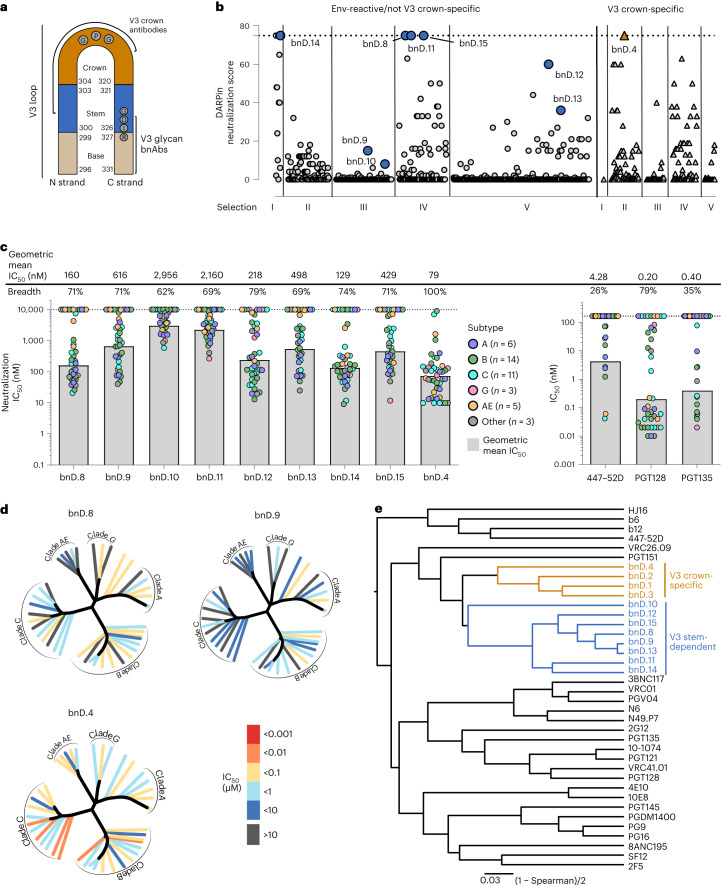
Identification of broadly neutralizing V3 inhibitors. **a**, Schematic of the V3 loop. Numbers indicate positions of delimiting amino acid residues of V3 base, V3 stem and V3 crown according to the HxB2 Env reference sequence. Brackets indicate binding sites of V3-targeting antibodies. **b**, Identification of bnDs targeting diverse sites in the V3 loop. Data depict basic neutralization breadth screening of DARPin clones isolated from high-diversity DARPin libraries in five independent ribosome display selections (I–V) with Env proteins and peptides as panning targets (Supplementary Fig. 1). Single clones (total, 948) from Env binder-enriched DARPin pools were screened for a distinct V3 binding pattern. Triangles depict V3 crown-reactive clones. Circles depict clones that do not bind to V3 crown peptides. Larger, blue circles denote V3 stem-dependent clones selected for follow-up. The large orange triangle denotes the V3 crown-specific clone bnD.4 selected for follow-up. Neutralization breadth and potency were assessed by a neutralization score against a 5-virus panel (Supplementary Table 2). **c**, Breadth and potency analysis of the nine selected bnDs (left) and reference bnAbs (right) on a 42-virus panel (Supplementary Tables 1 and 2). IC_50_ in nM is depicted for each virus, with virus subtype denoted by color. Data are geometric means from two or three independent experiments. Gray bars depict the geometric mean IC_50_ for all neutralized strains (IC_50_ < 10 µM). Breadth denotes the percentage of strains neutralized. **d**, Neutralization dendrograms of V3-specific bnD.8 and bnD.9 and V3 crown bnD.4 based on data depicted in **c** and Supplementary Table 2. Dendrograms show the Env diversity of the 42-virus panel. Branches are colored according to neutralization potency (IC_50_), and non-neutralized branches are in black. **e**, Neutralization fingerprinting of bnDs with V3 stem dependency specificity (blue), V3 crown bnDs (orange) and nAbs (black) based on data depicted in **c** and Supplementary Table 2. Dendrograms were generated by hierarchical cluster analysis (McQuitty’s method) based on dissimilarities of Spearman correlation of neutralization (Supplementary Table 2). Source data

**Extended Data Fig. 1:**
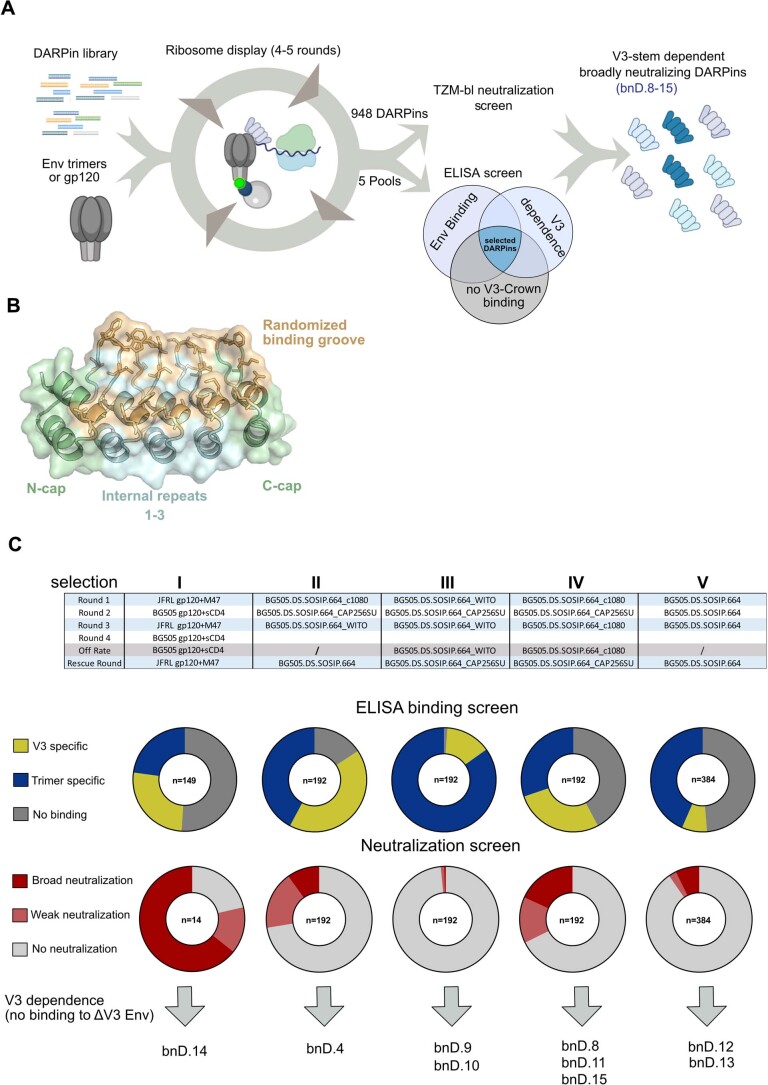
DARPin panning and screening. A) Set-up of DARPin ribosome display (RD) selection and screening to identify V3 specific broadly neutralizing DARPins (bnDs). B) Generic structure of a N3C-type DARPin with three internal ankyrin repeats (blue, with DARPin library randomized amino acid positions in gold) and amino- and carboxyterminal capping repeats (green). C) Overview of DARPin RD selections (I-V). Individual selection strategies dependent on the selection target were employed in successive RD panning rounds. BG505.DS.SOSIP.664_CAP256SU, _ c1080 and _WITO are BG505.DS.SOSIP.664 trimers with swapped V1V2 from strains CAP256SU, c1080_c03 and WITO4160cl33 based on previously described design^60^. Primary binding and neutralization screens were adjusted to match selection targets. DARPins binding six-fold over background to either BG505 V3 peptide or BG505.SOSIP.664 were categorized as specific Env binders. DARPins that bound BG505.SOSIP.664 but not V3-crown peptide and ΔV3.BG505.SOSIP.664 were ranked as V3 specific with epitopes extending beyond the V3-crown. DARPins with a neutralization score (see material and methods) below 5 were considered non-neutralizing. DARPins with a neutralization score of 5-14 were categorized as weakly neutralizing DARPins, DARPins with a score ≥15 as broadly neutralizing. Source data

**Extended Data Fig. 2:**
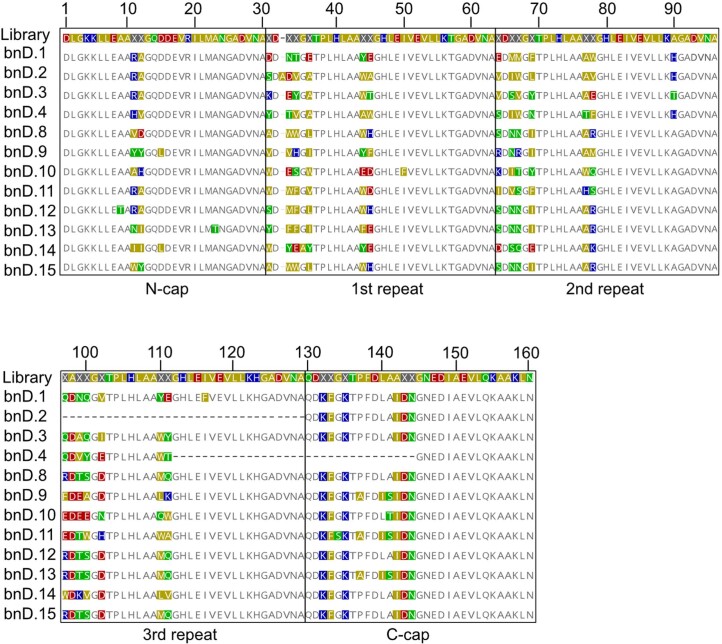
Alignment of V3 dependent bnDs. Amino acid sequence alignment of V3 bnDs selected in selections I-V (bnD.4 and bnD.8 to bnD.15) and in published V3 bnDs (bnD.1 to bnD.3^20^,. Alignment of V3-crown (bnD.1-bnD.4) and V3-CD4i bnDs (bnD.8-bnD.15). The library sequence indicates randomized residues by “X”, mutated residues in the respective DARPins are indicated by colors. All bnDs are N3C DARPins except bnD.2 and bnD.4 which are N2C DARPins lacking one internal repeat, indicated by dashes.

To identify neutralization-sensitive epitopes on HIV-1 Env, we leveraged highly diverse DARPin libraries. Env-binding DARPins were selected in five independent selection campaigns (selections I–V) using a range of Env trimers and monomers as panning targets (Extended Data Fig. 1a–c). Having recently shown that bnDs targeting the V3 crown can exhibit considerable breadth^20^, we specifically explored the potential of broad V3 neutralization beyond the V3 crown (Fig. 1a). To this end, a total of 948 DARPin clones were subjected to rigorous screens to identify clones of interest (Extended Data Fig. 1c). The criteria that had to be met included high neutralization breadth against a multiclade pseudovirus panel (*n* = 5) and the ability to bind Env trimer derivatives but not to a V3 crown peptide (Extended Data Fig. 1c). Interestingly, we observed a high fraction of DARPins with V3 reactivity across all selections (Extended Data Fig. 1c). This is in line with a proportion of even highly stabilized DS.SOSIP.664 trimers exposing the V3 (ref. ^28^), which may further increase owing to disassembly of trimers during ribosome display manipulations. Overall, the strong enrichment of V3-reactive clones underscores the high antigenicity of V3 in the DARPin system. To dissect the V3 crown and other V3 specificities among identified DARPins with breadth, reactivity to V3-deleted BG505.SOSIP.664 was assessed (Extended Data Fig. 1c). DARPins that bound to BG505.SOSIP.664 trimer but to neither the V3 crown peptide nor the V3-deleted BG505.SOSIP.664 were rated as V3 specific with epitopes extending to the V3 stem (referred to as V3 stem-dependent). Eight high-breadth DARPins met this specificity criterion (bnD.8–bnD.15; Fig. 1b) and were selected for follow-up experiments. In addition, one high-breadth V3 crown binding DARPin selected in these campaigns, clone bnD.4, was incorporated in further analyses for comparison (Fig. 1b). All nine DARPins were found to be distinct clones, unrelated to previously identified V3 crown-specific DARPins (Extended Data Fig. 2 (ref. ^20^)).

**Extended Data Fig. 3:**
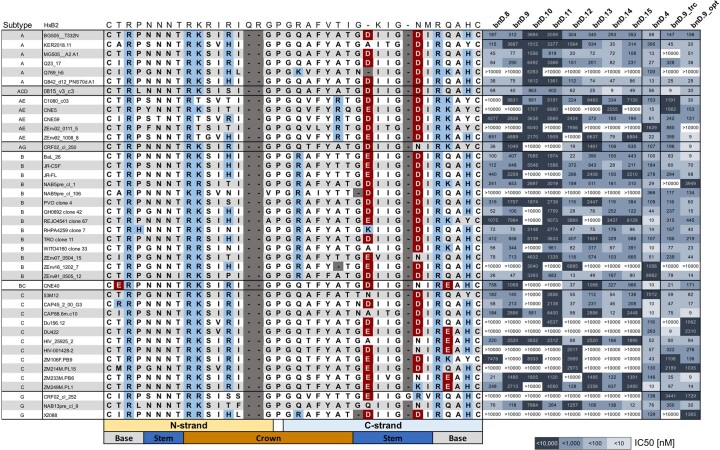
Alignment of V3 sequence in comparison to bnD IC50s. Sequence alignment of the V3 amino acid sequences of virus strains included in the 42-virus neutralization panel alongside their sensitivity to bnDs as determined in the TZM-bl assay. IC50 values (nM) are depicted.

Expanding the neutralization breadth analysis on a multiclade tier 2 HIV-1 pseudovirus panel (*n* = 42; Fig. 1c,d, Extended Data Fig. 3 and Supplementary Tables 1 and 2), we observed 100% breadth for the V3 crown-specific DARPin bnD.4, demonstrating the enormous potential of V3-directed neutralization. Notably, the group of V3 stem-dependent DARPins (bnD.8–bnD.15) likewise showed high neutralization breadth, inhibiting up to 79% of the strains tested (bnD.12), with the most potent (bnD.14) reaching a geometric mean potency against neutralized strains of 129 nM. In comparison, consistent with their known breadth and potency, the prototypical V3 crown monoclonal antibody (mAb) 447-52D reached only modest breadth of 26%, whereas bnAbs PGT128 and PGT135, which target the V3 base and associated glycans, achieved 79% and 35% breadth, respectively. Notably, although only PGT128 matched bnDs in breadth, antibodies were more potent than DARPins as previously observed for V3 crown bnDs^20^.

Neutralization profiling of bnDs and bnAbs revealed that V3 crown-specific bnD.4 clustered with previously identified V3 crown DARPins bnD.1, bnD.2 and bnD.3 (ref. ^20^) (Fig. 1e and Supplementary Table 2). However, bnDs with V3 stem dependency (bnD.8–bnD.15) formed a distinct group in the neutralization fingerprint analysis (correlating with each other, but not with known V3 crown bnDs or bnAbs). We conclude that bnDs with V3 stem dependency display a unique reactivity pattern and form a distinct inhibitor group (Fig. 1e).

### V3-CD4i bnDs engage an open, postattachment Env conformation

**Fig. 2:**
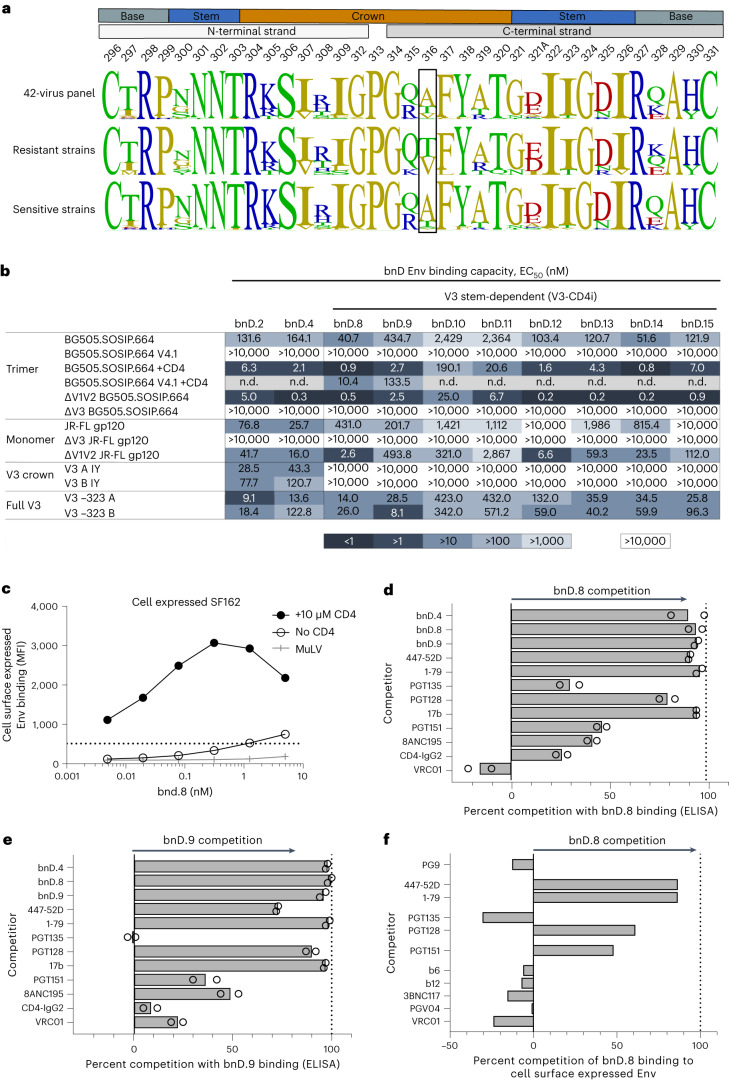
V3-CD4i bnDs target V3 C-terminal half on open Env. **a**, Distinct resistance pattern of bnDs with V3 stem dependency. Logo plots of V3 residues of 42-virus panel strains neutralized and resistant to bnD.8–bnD.15 based on data in Fig. 1c and Supplementary Table 2. Strains that were either neutralized or resistant to at least six of the eight bnDs were included in the analysis. Strains that contain deletions in V3 are not included. **b**, Binding properties of V3-reactive bnDs. Heat map depicts binding pattern to Env in the presence or absence of sCD4, Env mutants and peptides based on half-maximum effective concentration (EC_50_, in nM) in ELISA. If 50% binding was not reached at a maximum concentration of 5 μM, a value of >10,000 nM was recorded. Data are means of two or three independent experiments. **c**, Binding of bnD.8 to cell surface-expressed SF162 envelope. Binding was measured by flow cytometry. Env opening was induced by the addition of 10 µM sCD4. Data from one representative experiment out of two are shown. MFI, mean fluorescence intensity. **d**, Competition binding mapping in ELISA. Shown is the percent competition of binding of bnD.8 to ΔV1V2 BG505.SOSIP.664 by a competitor added at a concentration of 10 µg ml^−1^ (antibodies) or 10 µM (bnDs). Competition of 100% denotes full inhibition of bnD.8 binding compared with a no-competitor control. The mean from two independent experiments is shown, with open circles indicating replicates. **e**, Competition binding mapping in ELISA. Shown is the percent competition of binding of bnD.9 to ΔV1V2 BG505.SOSIP.664 by a competitor added at a concentration of 10 µg ml^−1^ (antibodies) or 10 µM (bnDs). Competition of 100% denotes full inhibition of bnD.9 binding compared with a no-competitor control. The mean from two independent experiments is shown, with open circles indicating replicates. **f**, Competition binding on cell surface-expressed ΔV1V2 SF162 Env. The binding of bnD.8 to Env was measured by flow cytometry after incubation with competitor. Competition of 100% denotes full inhibition of bnD.8 binding compared with a no-competitor control. Competition as observed at a competitor concentration of 10 µg ml^−1^ (antibodies) or 10 µM (bnDs) is depicted. No CD4 was added in this experiment. Data from one representative experiment out of two are shown. Source data

Sequence comparison of the V3 from resistant and sensitive strains provided insight into epitope features of V3 stem-dependent bnDs. Regardless of the HIV-1 subtype, resistance to these bnDs was mostly associated with a threonine or valine at V3 position 316 (Fig. 2a and Extended Data Fig. 3). In addition, strains containing a deletion in the C-terminal half of V3 were resistant to neutralization by V3 stem-dependent bnDs (Extended Data Fig. 3), suggesting a crucial contact site within the C-terminal half.

To gain further insight into bnD specificity, we expanded the initial enzyme-linked immunosorbent assay (ELISA) screen to a larger panel of Env derivatives and V3 peptides. bnD.4 bound V3 crown peptides well, confirming its specificity and close relationship with previously identified V3 crown-specific bnDs, such as bnD.2 (Fig. 2b). Consistent with their similar neutralization patterns, the eight bnDs with V3 stem dependency showed high similarity in binding to recombinant proteins and peptides in ELISA (Fig. 2b). They did not interact with V3 peptides representing solely the V3 crown (residues 305–320) but bound to full-length V3 peptides. V3 dependence was further confirmed by the inability to bind V3-deleted trimeric (ΔV3 BG505.SOSIP.664) and monomeric (ΔV3 JR-FL gp120) Env constructs (Fig. 2b). Binding to intact, stabilized trimers was weak at best. However, strong interactions were observed with the SOSIP.664 trimer after V1V2 deletion or trimer opening induced by soluble two-domain CD4 (sCD4) (Fig. 2b). The lack of binding was intriguingly pronounced for BG505.SOSIP.664 V4.1. This protein is highly stabilized and contains an A316W mutation in the V3 (ref. ^15^) that may further affect V3 DARPin binding. Nevertheless, bnD.8-bound and bnD.9-bound sCD4 triggered BG505.SOSIP.664 V4.1 efficiently. Results with cell surface-expressed Env trimers supported the dependence on CD4 opening, as bnD.8 showed only marginal binding unless the opening of the membrane-anchored trimer was triggered by sCD4 (Fig. 2c).

Focusing further analyses on bnD.8 and bnD.9 as representatives of the bnD group with V3 stem dependency, we observed in ELISA competition mapping a strong competition with V3 crown antibodies (1-79 and 447-52D), DARPin bnD.4 and PGT128 (Fig. 2d,e), as expected. Lower but still noteworthy competition (up to 50%) was also observed with the interface antibodies 8ANC195 and PGT151, possibly due to conformational fixation induced by these bnAbs. 8ANC195 was shown to limit conformational transitions to fully open Env, whereas PGT151 preferentially binds a closed or semiclosed Env conformation, which may influence the sampling of the open Env conformation recognized by bnD.8 and bnD.9 (refs. ^29–31^). Competition mapping with cell surface-expressed Env confirmed this pattern (Fig. 2f). Interestingly, we also observed strong ELISA binding competition of bnD.8 and bnD.9 with the CD4-induced (CD4i) epitope targeting antibody 17b, which interacts with the coreceptor binding site within gp120 upon CD4 triggering. Owing to their dependence on CD4 triggering and overlap with the CD4i mAb 17b, we designated the group of DARPins with V3 stem dependency represented by bnD.8 and bnD.9 as V3-CD4i bnDs.

**Fig. 3:**
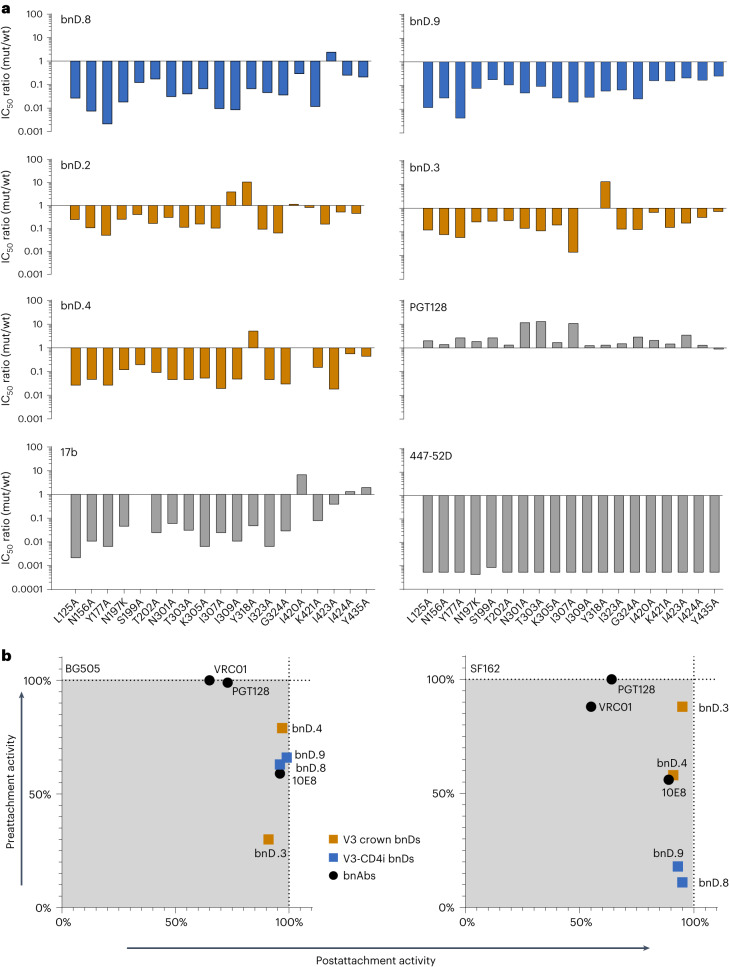
V3-CD4i bnDs are highly dependent on Env opening and act predominantly after attachment. **a**, Effect of trimer opening on the neutralization potency of bnD.8 and bnD.9. Single JR-CSF alanine mutants known to confer trimer opening were compared for sensitivity to bnDs and antibodies. Bars indicate fold change of IC_50_ relative to wild-type JR-CSF; depicted ratios are means from two to four independent experiments. mut, mutant; wt, wild-type. **b**, Preattachment and postattachment inhibition activity of bnDs and bnAbs against BG505 and SF162 pseudoviruses as deferred in an adapted TZM-bl pseudovirus assay where total activity corresponds to the conventional neutralization setup with inhibitor present at all steps and consisting of an activity before attachment and after attachment. Through measuring postattachment activity in a setup where inhibitors are added after virus binding to cells, preattachment activity can be deferred. This total activity measured at a fixed antibody (6.4 nM) or bnD (10 µM) concentration is set to 100%, and preattachment and postattachment activity are calculated relative to it. Values are means of three or four independent experiments. Source data

To further investigate the effect of trimer opening on neutralization by V3-CD4i bnDs, we used a panel of JR-CSF alanine mutants that we previously identified as having an open phenotype that confers high neutralization sensitivity to V3 crown antibodies such as 447-52D and V3 crown bnDs^12,20,32^. Compared with wild-type, these open JR-CSF mutants were up to 550-fold more sensitive to neutralization by the V3-CD4i bnDs bnD.8 and bnD.9, resembling the effect on the CD4i mAb 17b (Fig. 3a and Supplementary Table 3). V3 crown antibody 447-52D showed the highest sensitivity gain over wild-type, while V3 glycan bnAb PGT128 showed no alteration or a modest loss in activity. Compared with V3-CD4i bnDs, the V3 crown bnDs (bnD.2–bnD.4) benefited less from trimer opening, highlighting a differential mode of action (Supplementary Table 3). Considering that V3-CD4i bnDs effectively inhibit tier 2 viruses in conventional neutralization assays (Fig. 1), their strong dependence on trimer opening is surprising and implies that their main activity must be in blocking the entry process after virus attachment to target cells. Therefore, we measured the neutralizing activity of bnD.8 and bnD.9 before and after attachment against the closed tier 2 strain BG505 and the open tier 1 strain SF162 (Fig. 3b)^17,33,34^. In contrast to bnAbs PGT128 and VRC01, the membrane proximal external region (MPER) bnAb 10E8 as well as V3-CD4i and V3 crown bnDs showed a clear predominance of activity after attachment. Although the tier 1 strain SF162 is known to have an open, neutralization-sensitive Env, epitope exposure was not sufficient to allow effective neutralization by bnD.8 and bnD.9 prior to CD4 binding. However, once SF162 had bound to host cells, a substantial increase in neutralization was observed, exceeding 90% of the total inhibitory activity.

### V3-CD4i bnDs define an α-helical V3 conformation

**Fig. 4:**
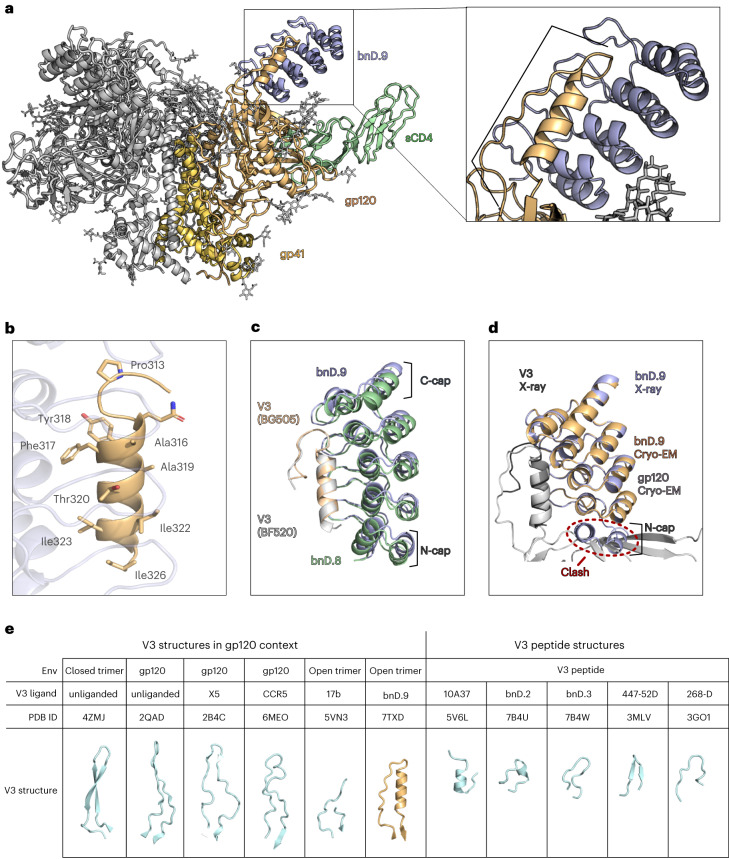
Structure definition of V3-CD4i bnD V3 binding. **a**, Cryo-EM structure at 3.9 Å resolution of bnD.9 in complex with BG505.SOSIP.664 trimer induced by sCD4. For one protomer, gp41 (yellow), gp120 (orange), bnD.9 (light blue) and sCD4 (light green) are indicated. **b**, Helical V3 segment as seen in the X-ray structure of the bnD.9–V3 (BG505) peptide complex. V3 interface residues that are within 4 Å distance to bnD.9 are shown as sticks. **c**, Comparison of X-ray structures of bnD.8 in complex with V3 (BF520) and bnD.9 in complex with V3 (BG505) peptide. The structures were superimposed on V3 in PyMOL. bnD.9 is shown in blue, with the bound V3 peptide in orange. bnD.8 is depicted in green, with its bound V3 peptide in gray. **d**, Superposition of bnD.9–V3 (BG505) X-ray structure and bnD.9–BG505.SOSIP–sCD4 cryo-EM structure (superposition based on V3). **e**, Comparison of bnD.9-bound V3 with published V3 structures. PDB IDs are indicated above the respective structures. From left to right: closed, unliganded trimer^49^; HIV-1 YU2 gp120 in complex with 412D and sCD4 (ref. ^50^); HIV-1 JR-FL gp120 in complex with sCD4 and X5 antibody^51^; gp120 in complex with the CCR5 and sCD4 (ref. ^35^); not fully resolved V3 on open trimer in complex with 17b and sCD4 (ref. ^23^); bnD.9-bound V3 on sCD4-triggered open trimer (this work); 10A37-bound peptide^36^; bnD.2-bound peptide^20^; bnD.3-bound peptide^20^; 447-52D-bound peptide^52^; and 268-D-bound MN peptide^52^.

**Extended Data Fig. 4:**
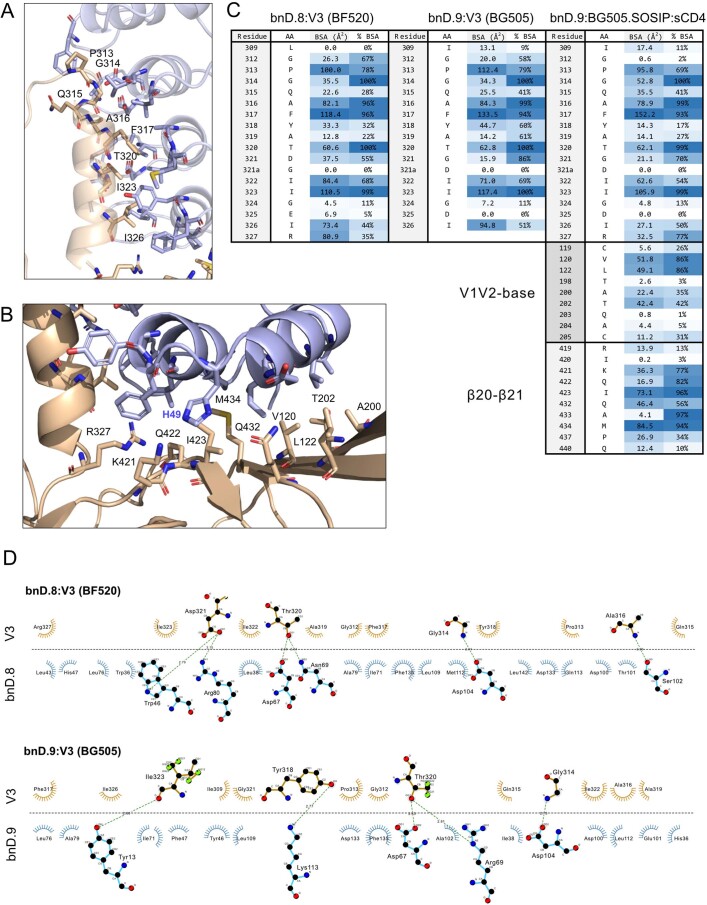
Interface of bnDs and Env. A) Interface of bnD.9 and V3 on BG505.SOSIP.664. Residues within 4 Å distance between bnD.9/Env are labeled and shown as sticks. B) Interface of bnD.9 and bridging sheet. Residues within 4 Å distance between bnD.9/Env are labeled and shown as sticks. C) Buried surface Area (BSA) of V3 residues and additional sites in β20-β21 and the V1V2-base in bnD.8 and bnD.9 X-ray structures in complex with V3 peptides and bnD.9 cryo-EM structure in complex with BG505.SOSIP.664 and sCD4. The % BSA was calculated as the ratio of BSA to accessible surface area. D) Ligplot depiction of DARPin/Env interface in X-ray structures of bnD.8 and bnD.9. DARPin residues are shown in blue, Env residues in orange. Eyelashes indicate a hydrophobic interaction, dotted green lines hydrogen bonds.

**Extended Data Fig. 5:**
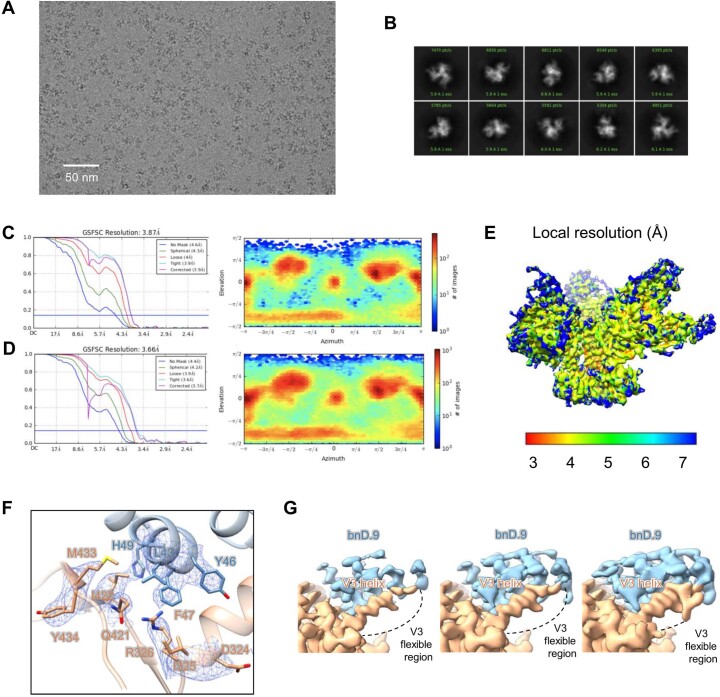
Cryo-EM details of bnD.9 in complex with BG505.SOSIP.664 and sCD4. A) Representative cryo-EM micrograph of bnD.9 in complex with BG505.SOSIP.664 and sCD4. Representative micrograph, of 2813 collected micrographs, is shown. B) Representative 2D class averages of bnD.9 in complex with BG505.SOSIP.664 and sCD4. C) The gold-standard Fourier shell correlation resulted in a resolution of 3.87 Å for the overall map using non-uniform refinement (left panel); the orientations of all particles used in the final refinement are shown as a heatmap (right panel). D) The gold-standard Fourier shell correlation resulted in a resolution of 3.66 Å for the locally refined map obtained using a mask that included bnD.9 and the interacting region of gp120 (left panel); the orientations of all particles used in the final refinement are shown as a heatmap (right panel). E) The local resolution of the final overall map is shown, generated through ResMap. F) Representative density is shown for the interface between bnD.9 and gp120; the contour level is 0.48 (1.2σ). BnD.9 is colored in light blue, gp120 subunit is colored in orange. Electron density of the V3 helix is shown at different contour levels: 0.25 (7.8 s, left), 0.3 (9.4 s, middle), 0.35 (10.9 s, right). The flexible region not visible in the map is represented as a dashed line.

To elucidate the neutralization mode underlying their high Env opening dependence, we performed structural analyses of V3-CD4i bnDs in complex with V3 peptides and trimeric Env. Crystals of bnD.8 and bnD.9 in complex with linear BF520 and BG505 V3 peptides diffracted to a resolution of 1.22 Å and 1.17 Å, respectively, enabling a detailed analysis of the DARPin–V3 interface and definition of contact residues (Fig. 4, Table 1 and Extended Data Fig. 4). To explore binding in the context of the open Env trimer, we determined the cryo-EM structure of open spike (native-like BG505.SOSIP.664 triggered by sCD4) in complex with bnD.9, which resolved to a resolution of 3.9 Å (Fig. 4a, Table 2 and Extended Data Fig. 5). Strikingly, in both the crystal structures and the cryo-EM structure, V3 formed a distinct, highly similar secondary structure when complexed with the bnDs: a four-turn α-helix extending from residues G314 to I323 (Fig. 4a–c). We termed this unique four-turn α-helix ‘αV3C’. In both the complex with bnD.8 and the complex with bnD.9, one face of the α-helix, including the ^312^GPG^314^ turn, was buried in the DARPin binding groove (Fig. 4b,c). A similar extended α-helix within V3 has not been observed previously. In contrast, V3 was mostly found to adopt a β-hairpin structure in the context of closed Env trimer and in complex with a range of antibodies (Fig. 4e). Molecular dynamics simulations of the V3 crown also suggested that β-hairpin structures dominate V3 conformation^20^. Interestingly however, a partially helical V3 crown conformation is bound by the coreceptor CCR5 (ref. ^35^), as well as two V3 crown-specific ligands: rabbit antibody 10A37 (ref. ^36^) and DARPin bnD.2 (Fig. 4e). Notably, these helices are shorter and do not extend to the V3 base as observed for αV3C in complex with the V3-CD4i bnDs.

**Table 1:** Cryo-EM data collection, refinement and validation statistics

	bnD.9 in complex with BG505.SOSIP (EMD-26157), (PDB 7TXD)
**Data collection and processing**	
Magnification	×81,000
Voltage (kV)	300
Electron exposure (e–/Å^2^)	41.92
Defocus range (μm)	–0.8/–2.5
Pixel size (Å)	1.07
Symmetry imposed	C1
Initial particle images (no.)	406,207
Final particle images (no.)	115,325
Map resolution (Å)	3.87
FSC threshold	0.143
Map resolution range (Å)	3.8–20
**Refinement**	
Initial model used (PDB code)	5VN3
Model resolution (Å)	3.93
FSC threshold	0.143
Map sharpening *B* factor (Å^2^)	119.2
Chimera CC	0.83
EMRinger score	1.64
Model composition	
Non-hydrogen atoms	20,555
Protein residues	2,483
Ligands	95
*B* factors (Å^2^), mean	
Protein	107.62
Ligand	93.42
R.m.s. deviations	
Bond lengths (Å)	0.005
Bond angles (°)	4.51
**Validation**	
MolProbity score	1.54
Clashscore	4.51
Poor rotamers (%)	0
Ramachandran plot	
Favored (%)	95.5
Allowed (%)	4.5
Disallowed (%)	0

**Table 2:** Data collection and refinement statistics (molecular replacement)

	bnD.8–V3 (BF520) (PDB 7Z7C)	bnD.9–V3 (BG505)( PDB 8AED)
**Data collection**		
Space group	C2	P2_1_
Cell dimensions		
* a*, *b*, *c* (Å)	96.22, 40.60, 45.63	50.79, 55.23, 58.03
α, β, γ (°)	90.0, 113.90, 90.0	90.0, 98.54, 90.0
Resolution (Å)	41.72–1.22	57.39–1.17
	(1.27–1.22)	(1.25–1.17)
*R*_sym_ or *R*_merge_	0.057 (1.055)	0.071 (0.562)
*I* / σ*I*	13.0 (1.5)	12.3 (1.8)
Completeness (%)	95.2 (90.0)	74.6 (20.4)
Redundancy	6.8 (6.9)	6.2 (3.5)
**Refinement**		
Resolution (Å)	1.22	1.17
No. reflections	45,596 (4,269)	79,873 (1,718)
*R*_work_ / *R*_free_	0.147 (0.536) / 0.196 (0.493)	0.154 (0.258) / 0.184 (0.272)
No. atoms	1,562	3,411
Protein	1,397	2,865
Ligand/ion	12	93
Water	153	493
*B* factors	30.81	15.61
Protein	29.33	13.16
Ligand/ion	34.93	19.63
Water	43.98	29.43
R.m.s. deviations		
Bond lengths (Å)	0.017	0.005
Bond angles (°)	1.87	0.84

Values in parentheses are for highest-resolution shell.

X-ray crystallographic and cryo-EM structure analyses of V3-CD4i bnD bound to V3 showed a high agreement (Fig. 4c,d). The DARPins interacted, via their variable, randomized repeat regions, with αV3C (Fig. 4c and Extended Data Figs. 1b and 4). The binding interface accessed by DARPins comprised residues bridging almost the entire C-terminal half of V3. The most buried residues include P313, G314, A316, F317, T320 and I323 (Extended Data Fig. 4c). Both bnD.8 and bnD.9 binding are facilitated by several hydrogen bonds with most relevant sites shared; for both DARPins, T320^V3^ interacted with DARPin residues D67 and N/R69 (bnD.8/9), and G314^V3^ bonded with DARPin residue D104. Bonds between D321^V3^ and A316^V3^ with DARPins W46/R80 and S102, respectively, are unique in bnD.8, whereas Y318^V3^ with K113 and I323^V3^ with Y13 are unique to bnD.9 (Extended Data Fig. 4d).

**Extended Data Fig. 6:**
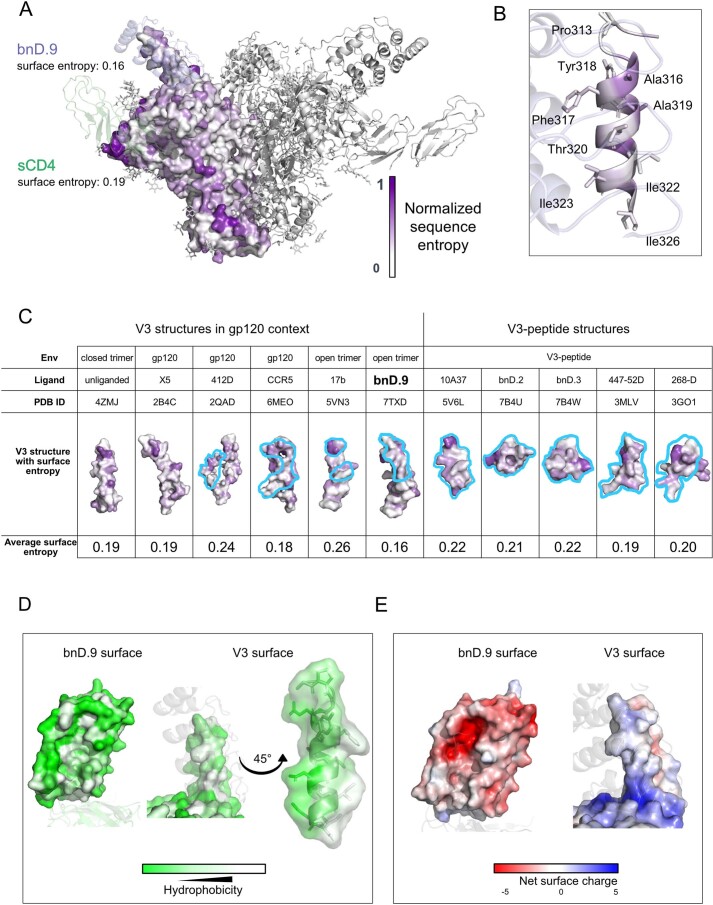
Analysis of the V3-CD4i bnD – gp120 interaction surface. A) Entropy of the bnD.9 binding area. Sequence diversity was calculated as normalized entropy based on the 42-virus panel and plotted on one protomer. Dark purple indicates high diversity. Binding residues were defined as residues with non-zero buried surface area (calculated by NACCESS) and the average entropy of the bnD.9 binding area on gp120 calculated as 0.16, the entropy of sCD4 binding area as 0.19. B) Normalized sequence entropy on helical V3 as defined by X-Ray structure of the bnD.9:V3 (BG505) complex across the 42-virus panel colored for diversity as in (A). V3 interface residues that are in 4 Å distance to bnD.9 are shown as sticks. C) Normalized sequence entropy of surfaces on V3 recognized by CCR5 co-receptor, bnDs and antibodies across the 42-cross-clade virus panel (Supplementary Table S1) is indicated by a color gradient as in A. V3-binding areas of ligands are encircled in cyan. For structures describing epitopes of antibodies and CCR5 extending beyond V3 the average entropy was calculated for the binding area on V3 only. D) Hydrophobicity of bnD.9 and V3 surfaces. Analysis was performed based on the cryo-EM structure of bnD.9:BG505.SOSIP:sCD4. Hydrophobicity scores are based on the Eisenberg hydrophobicity scale^102^ and depicted as a gradient, with hydrophobic residues shown in white. E) Surface charge of DARPin and V3 on gp120. Analysis was performed based on the cryo-EM structure of bnD.9:BG505.SOSIP.664:sCD4. Surface charge was calculated using the APBS electrostatics PyMOL plugin and visualized using a color gradient.

Remarkably, the Env surface recognized by bnD.9 is as conserved as the CD4 binding site (average sequence entropy of contact residues for 7,590 group M strains listed in the National Institutes of Health (NIH) sequence database, 0.22 (bnD.9) and 0.21 (CD4 binding site); for strains in the 42-virus panel, 0.16 (bnD.9) and 0.19 (CD4 binding site); Extended Data Fig. 6a–c and Supplementary Fig. 2). This comparatively low sequence entropy of V3 contact residues for bnD.9 (Extended Data Fig. 5b) may provide an explanation for the exceptional breadth of bnD.9.

Analysis of bnD.9 positioning in complex with V3 peptide by X-ray crystallography (Fig. 4b), and on the sCD4-triggered trimer by cryo-EM (Fig. 4a,d), revealed an intriguing aspect on the binding mode. The N cap (amino-terminal ankyrin capping repeat) of bnD.9 is clearly visible in the crystal structure of the V3 peptide complex, but no defined corresponding electron density was observed in the trimer complex, suggesting that N cap displacement may contribute to epitope accessibility (Fig. 4d). Indeed, N cap truncation can increase potency of V3-CD4i bnDs (Supplementary Note). Overall, the αV3C proved to be an amphipathic helix, with the hydrophobic face oriented toward the likewise hydrophobic DARPin binding groove, thereby allowing multiple hydrophobic interactions to contribute to binding (Extended Data Fig. 6d). In the N cap-displaced bnD.9 structure, these interactions also extend to the interface with the gp120 bridging sheet. Additional contributions come from charge interactions between negatively charged DARPin residues and positive charges on V3 (Extended Data Fig. 6e).

### Accessibility of α-helical V3

**Extended Data Fig. 7:**
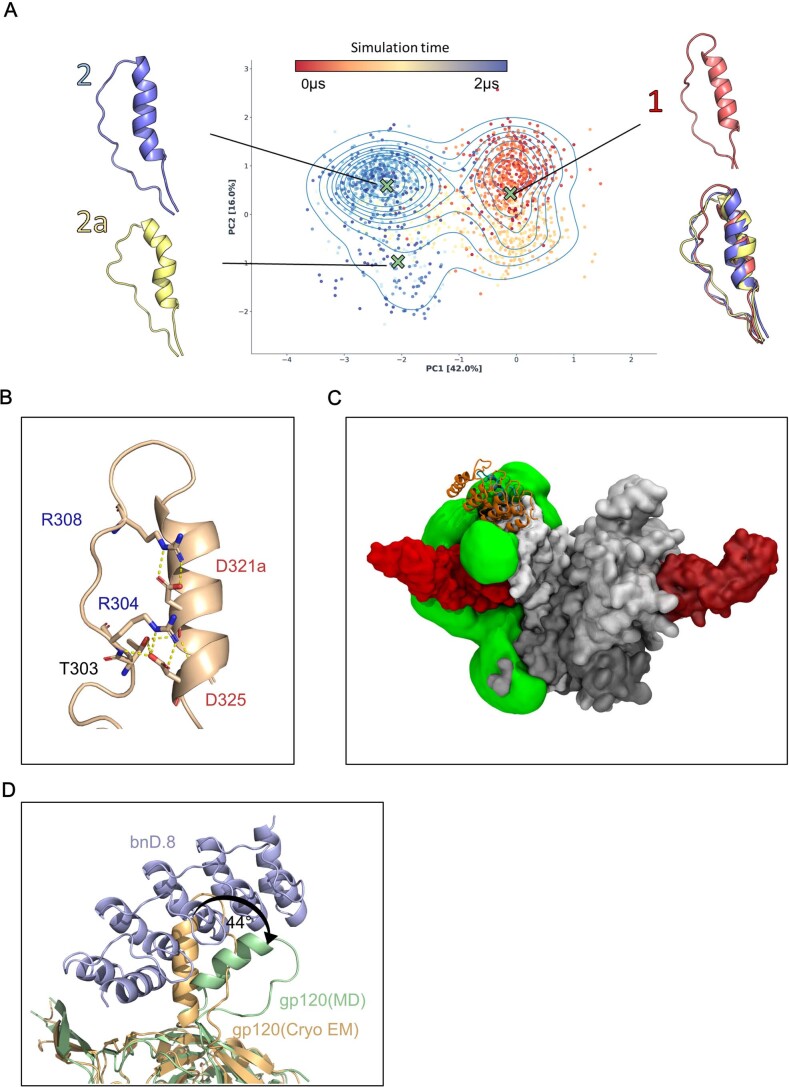
Molecular Dynamics simulation of gp140 without bnD.9. A) Principal component analysis (PCA) of αV3C dynamics. Each point represents a conformation sampled during the MD simulation starting from the bnD.9:BG505.SOSIP.664:sCD4 cryo-EM structure (0µs, red) up to 2μs of simulation (blue) in absence of sCD4. Contour lines depict the frequency of adopted αV3C conformations. Green crosses indicate the centroids defined by a k-means clustering, and the corresponding V3 structures are shown as cartoons. B) Stick representation of the salt bridges stabilizing the helical V3 during the MD simulation. C) Glycan-density (green surface) covering the Env surface during the 2µs MD simulation time on one protomer (gp120 in light grey, gp41 dark grey, V3 dark blue and sCD4 in red). The simulation was done in absence of bnD.9. For display, bnD.9 (orange) was docked to helical V3 of the final frame of the simulation. D)Molecular docking of bnD.8 onto one frame of an accessible conformation of the helical V3 sampled during the MD simulation.

The fact that we selected several inhibitors with similar reactivity pattern from independent selections suggests that the αV3C conformation defined in the complexes with DARPins must be populated to a substantial degree, consistent with a transient Env intermediate conformation that forms spontaneously and during the entry process. We employed molecular dynamics simulations using the cryo-EM structure of BG505.SOSIP.664 obtained in complex with bnD.9 to probe the stability of the αV3C-helix. Molecular dynamics simulations showed that the helix remained stable in the absence of bnD.9 for up to 2 µs of the simulation and extended further N terminally to residue P313 during this time frame (Extended Data Fig. 7a). Helix-stabilizing salt bridges formed between N- and C-terminal half residues, R304 and D325 and R308 and D321 (Extended Data Fig. 7b). Of note, molecular dynamics simulations showed that the αV3C-helix was not shielded by glycans, indicating possible accessibility (Extended Data Fig. 7c).

**Extended Data Fig. 8:**
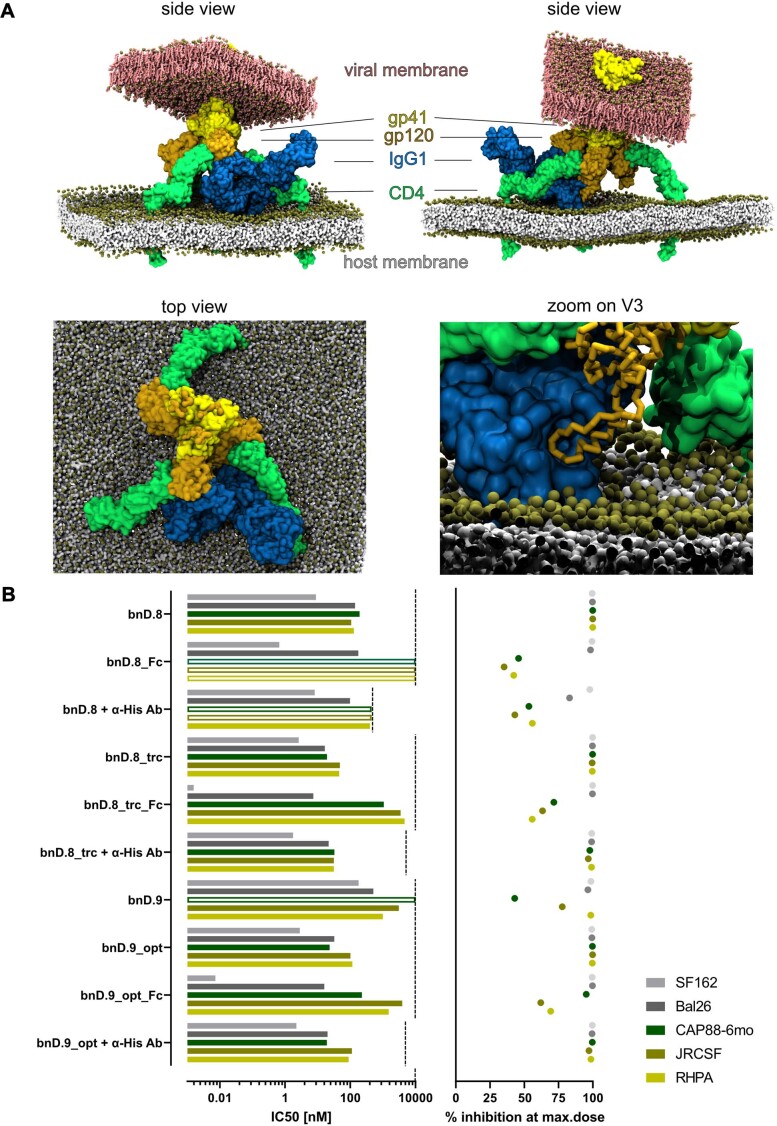
Molecular model of the αV3C accessibility to an IgG antibody. A) Molecular model showing an IgG antibody engaging with the HIV-1 Env and CD4 complex as seen from the side (top) and from the virus (bottom left). All proteins are represented by their molecular surface, with gp41 colored in yellow, gp120 in orange, CD4 in green, and IgG in blue. The hydrophobic core of the viral and T-cell membranes is shown with pink and light grey sticks, respectively, and the phosphate groups are highlighted with gold spheres. B) Neutralization capacity of size-increased variants of bnD.8 and bnD.9. DARPin-Fc fusion constructs are bivalent constructs of DARPin fused at its C-terminus to the constant region of huIgG1. bnD + α-His Ab are complexes of the bnD with an anti-His antibody bound non-covalently to the DARPin’s N-terminal His-Tag. Bars on the left depict IC50 against the indicated Env pseudotyped virus strains. The dotted line indicates the maximum assay concentration, empty bars indicate that 50% inhibition was not reached at this concentration. Dots on the right depict percent inhibition reached at the maximum concentration of each inhibitor. Values from one of two independent experiments are shown. Inhibitors were titrated from 2.15 μM (bnD.8_trc + α-His Ab), 2.07 μM (bnD.9_opt + α-His Ab), 0.45 μM (bnD.8 + α-His Ab) and 10 μM for all other inhibitors. Source data

The N cap displacement observed with bnD.9 (Fig. 4d) suggested constraints in steric accessibility of αV3C, which we confirmed by creating N cap truncated versions of bnD.8 and bnD.9 (Supplementary Fig. 3 and Supplementary Note). Although antibodies have flexible binding regions that can provide opportunities over DARPins to reach into small cavities, the overall larger size of antibodies may be access-limiting. Indeed, antibodies binding to the CD4i region were found to access their epitopes better as antigen-binding fragments (Fabs) than as full antibody^37^. To address this observation in the context of αV3C, we conducted a coarse-grained molecular dynamics simulation that allowed for estimation of the spatial accessibility for full antibody molecules to Env bound to three CD4 molecules in a membrane context (Extended Data Fig. 8a). Notably, our analysis confirmed that IgG molecules, in principle, could access αV3C on CD4-bound trimer (Supplementary Note).

### Escape from V3-CD4i bnDs preserves V3 conformation

**Fig. 5:**
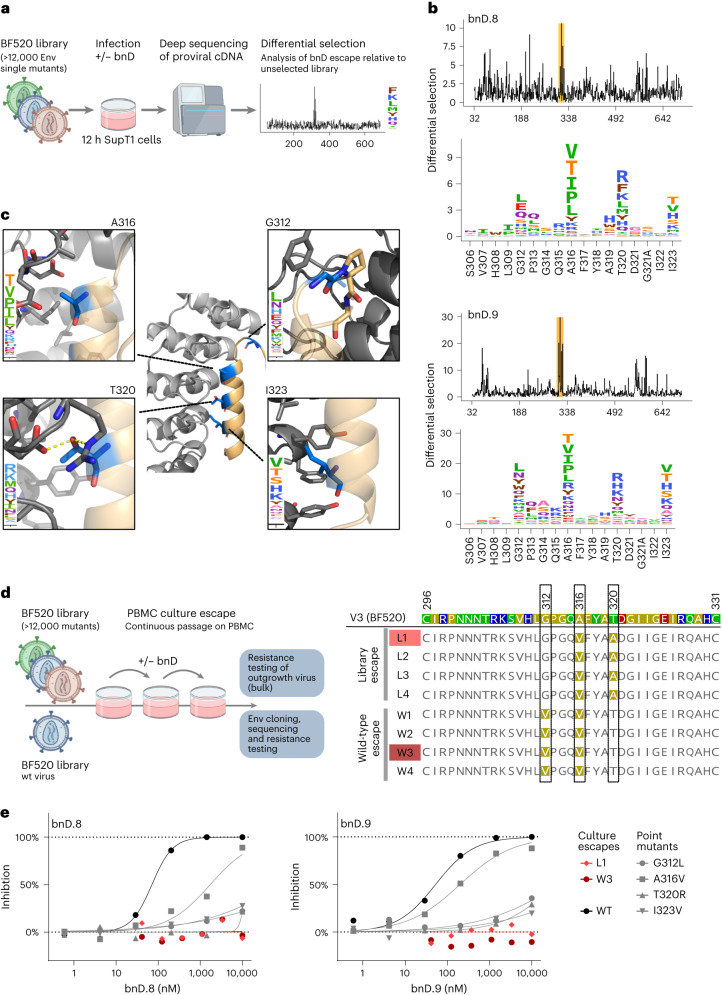
Mutational antigenic profiling defines V3-CD4i bnD epitope and escape. **a**, Mutational antigenic profiling of bnD.8 and bnD.9 resistance. **b**, Line plots (top panels) depicting results of mutational profiling. Lines indicate the differential selection, which is the logarithm of the relative enrichment of mutations in the BF520 mutant library upon neutralization with bnD.8 (0.56 µM) or bnD.9 (3 µM) averaged across all mutations at each site (Extended Data Fig. 10a). The V3 region is highlighted by a yellow shaded area. Bottom panels depict V3 profiles as sequence logo plots. The height of the letters is proportional to the differential selection of respective amino acids. Mean differential selection of two independently generated and selected BF520 mutant libraries is shown. **c**, Escape mutations localize along the DARPin–V3 helix interface. Visualization of highly selected resistance sites defined in **b** on the X-ray structure of bnD.9 in complex with V3 (BG505) peptide. Zoomed images depict selected residues and their surrounding areas. **d**, Continuous escape cultures of BF520 mutant library and BF520 wild-type virus on PBMC in the presence of escalating doses of bnD.8 (Extended Data Fig. 10). V3 sequence of escape strains isolated aligned to the BF520 wild-type sequence. **e**, Resistance testing of BF520 mutants defined in **b**, **c** and **d** in the pseudovirus TZM-bl assay against bnD.8 and bnD.9. Data depict means from two independent experiments. Source data

With V3-CD4i bnD binding sites in V3 defined, we sought to elucidate the functional importance of distinct V3 residues and potential escape pathways. Given the high dependence of V3-CD4i DARPins on the opening of the trimer and the formation of the αV3C-helix, we hypothesized that mutations affecting helix conformation and trimer stability, in addition to changes in the actual contact region, might promote escape. To define escape pathways, we used a sequencing-based mutational antigenic profiling approach based on a comprehensive single point Env mutant virus library based on the CCR5 tropic strain BF520 (refs. ^38,39^) (Fig. 5a).

We observed escape mutations along the entire Env ectodomain (Fig. 5b), with the most enriched escape sites for both bnD.8 and bnD.9 located in V3, with G312, A316, T320 and I323 showing the strongest differential selection for both DARPins (Fig. 5b). Several of these sites form the interface of αV3C and the DARPin binding groove (Fig. 5c) and are also among the residues with the most buried surface area (Extended Data Fig. 4c). An exception is G312, which, based on structural data, does not contribute substantially to the interaction with the bnDs. However, substitution of the flexible glycine likely affects the conserved GPG turn motif and thus alters the secondary structure of the V3. T320 forms two hydrogen bonds with bnD.8 and bnD.9 that seem to be critical for bnD docking, as identified escape mutations fail to form hydrogen bonds with the DARPins.

Two escape sites, A316 and I323, are buried deep in the hydrophobic interface, rendering replacement by bulkier residues prone to disruption of the contact surface. The overall strongest differential selection occurred at A316 (Fig. 5b). A316V and A316T identified by mutational scanning were also detected among resistant viruses in the neutralization fingerprint analysis (Fig. 2a and Extended Data Fig. 3). A316W has been described to reduce V3 exposure^16^, indicating a possible additional influence of mutations at this site on V3 accessibility. Interestingly, another residue buried deep in the interface, F317, was not subject to escape. This residue is highly conserved and bears little tolerance for mutations^39^. Although Y318 forms a hydrogen bond with bnD.9, no escape was observed. While Y318 is highly conserved in vivo, it is tolerant for mutations in the BF520 Env mutant library^39^. Collectively, this suggests that a Y318 bond is not critical for bnD.9 binding. Considering the negatively charged DARPin binding surface, the introduction of negative charges within V3 may seem a likely escape path. However, we observed only sporadic negatively charged escape residues, consistent with the fact that it is functionally relevant for the V3 loop to retain positively charged residues for the interaction with the coreceptors. Of note, prolines that would be expected to interfere with the formation of αV3C were equally rare among the escape mutations, appearing only as minor variants at site 316 at the N terminus of αV3C, at a position where proline can be tolerated^40^. Overall, the observed escape mutation patterns are consistent with a strong pressure to maintain V3 characteristics. This was further supported by a survey of the HIV Sequence Database (https://www.hiv.lanl.gov), which confirmed that mutations in the identified V3-CD4i escape sites are rarely observed in circulating HIV-1 strains (Supplementary Fig. 4).

**Extended Data Fig. 9:**
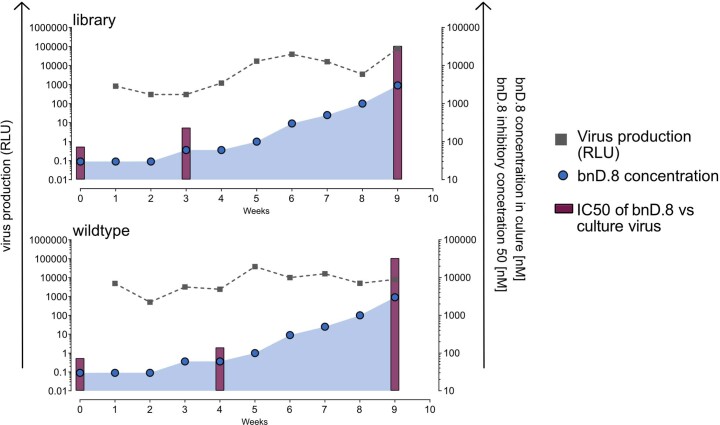
PBMC culture escape virus selection. Representative profile of in vitro escape selection on PBMC performed with bnD.8. Panels depict the evolution of the resistant viral variants of BF520, starting with the infection of BF520 mutational library virus mixture (top) or the BF520 wildtype isolate (bottom). Grey dotted lines depict the virus production in culture supernatant measured as relative light units (RLU) on TZM-bl reporter cells. Shaded blue areas depict increasing bnD.8 concentration supplied during the selection culturing. Bars depict neutralization sensitivity (IC50) of the polyclonal virus mix in culture supernatant retrieved at the indicated time points. Source data

To verify these results, we performed escape selection experiments with replication-competent virus in peripheral blood mononuclear cell (PBMC) cultures (Fig. 5d and Extended Data Fig. 9). In two parallel experiments, escape to bnD.8 was studied using either BF520 wild-type virus or the BF520 mutant library virus cocktail used in comprehensive mapping as inoculum. Dose escalation of bnD.8 over 9 weeks of culture led in both cultures (BF520 wild-type and library) to the emergence of escape mutations in the same region identified by mutational scanning. However, single mutations appeared to be insufficient to manifest escape in multiple-round infection of PBMC. Identified escape variants introduced the mutation A316V in combination with either T320A (library) or G312V (wild-type) (Fig. 5d).

To further elucidate the effect of key escape mutations, we assessed inhibitor susceptibility of single point mutant BF520 pseudovirus and escape strains isolated in the PBMC culture escape in a conventional TZM-bl inhibition assay (Fig. 5e). A316V failed to confer complete resistance but still led to a marked shift in potency. G312L, T320R and I323V rendered the virus highly resistant to inhibition by bnD.8 and bnD.9, confirming the critical contribution to these residues in binding. Double mutations identified in PBMC culture escapes (G312V + A316V and A316V + T320A) were fully resistant to both bnDs. Notably, among the identified resistance mutation combinations, only the A316V + T320A double mutation has been previously described in naturally occurring strains, albeit at low frequency (11 out of 5,022 group M strains listed in the NIH sequence database).

### Escape from V3-CD4i bnDs limits trimer opening

**Fig. 6:**
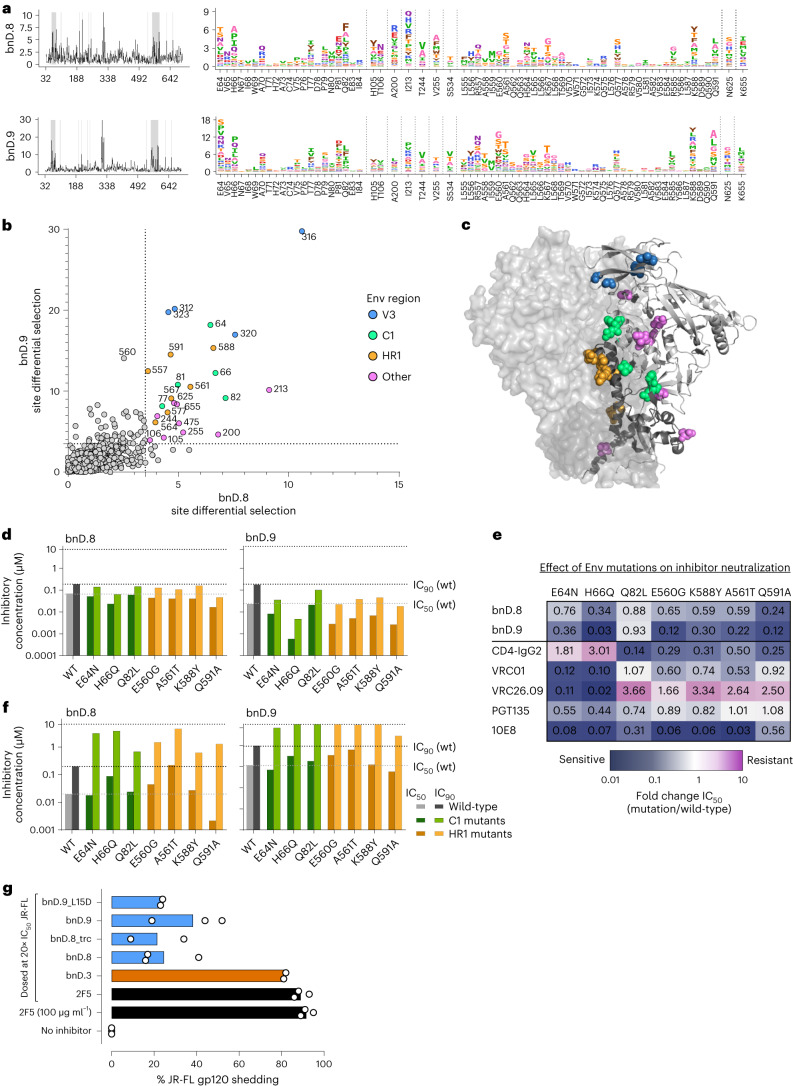
Secondary site mutations influence Env stability and pliability. **a**, Mutational antigenic profiling of bnD.8 and bnD.9 resistance as outlined in Fig. 5 focusing on regions outside V3. Line plots (left panel) indicate differential selection. Env regions shaded in gray are shown as logo plots in the right panel. Mean differential selection of two independently selected BF520 libraries is shown. **b**, Comparison of mutational antigenic profiling of bnD.8 and bnD.9 shown in Fig. 5b and **a** based on site differential selection readout (circles). Sites with differential selection >3.5 shared by bnD.8 and bnD.9 within V3 (blue), C1 (green), HR1 (orange) or other regions (purple) are highlighted. **c**, Localization of differentially selected sites shared by bnD.8 and bnD.9 (shown in **b**) on JR-FL Env (PDB 5FUU). Clusters in V3 (blue), C1 (green) and HR1 (orange) are highlighted. Other sites are shown in purple. **d**, Functional analysis in the conventional TZM-bl assay of BF520 resistance sites outside V3 detected in mutational scanning (**a**–**c**) shows heightened sensitivity to bnD.8 and bnD.9. **e**, Env mutations analyzed in **d** predominantly increase sensitivity to neutralizing antibodies underlining effects on Env stability and quaternary structure. The ratio of IC_50_ values derived in the conventional TZM-bl assay for mutant/wild-type BF520 virus is shown. **f**, Modified TZM-bl assay recapitulating bnD.8 and bnD.9 resistance patterns defined during deep mutational scanning after harmonization of culture conditions. Removing inhibitors and unbound pseudovirus (BF520 wild-type and mutants) after 3 h co-incubation with cells (analogous to conditions during mutational antigenic profiling) shows decreased sensitivity of mutants to bnD.8 and bnD.9. **g**, Induction of gp120 shedding from JR-FL Env-pseudotyped virions by bnAb 2F5 (black), the V3 crown-directed bnD.3 (orange), and V3-CD4i bnD versions (blue). Shedding activity is depicted as percent gp120 shedding relative to the mock-treated control, normalized to p24 levels. Concentration of inhibitors was adjusted to 20× above the respective IC_50_. 2F5 was tested in addition at 100 µg ml^−1^. Bars depict mean values from two (bnD.9_L15D, bnD.8_trc, bnD.3) or three (all other inhibitors) independent experiments shown as open circles. bnD.8_trc is a more potent variant of bnD.8 that lacks the N cap; bnD.9_L15D is a more potent point mutation variant of bnD.9 (see Supplementary Note and Supplementary Fig. 3). Source data

In addition to mutations directly affecting binding, we observed extensive differential selection at sites outside V3 (Fig. 6a). Interestingly, of the 20 most frequently selected sites outside of V3, 19 are located at the protomer or subunit interface (Fig. 6b). Mutations notably accumulated in the C1 region of gp120 and in the HR1/CC loop in gp41. These two regions are of high importance for the stability of Env and conformational transitions^23,41–44^. Remarkably, one of the strongest selection pressures was exerted on the region that forms the α0-helix upon opening of the trimer^23^.

**Extended Data Fig. 10:**
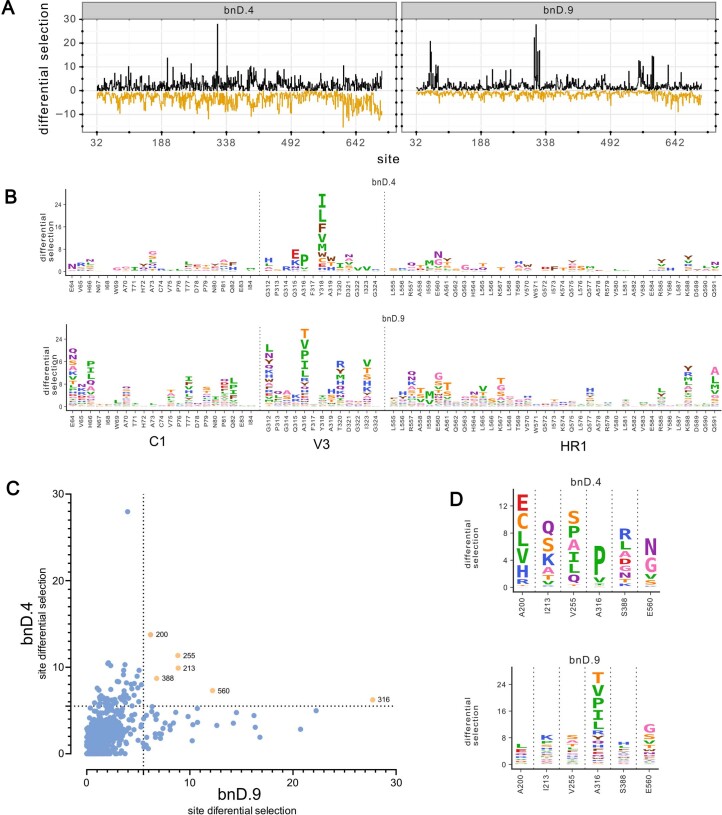
Mutational antigenic profiling of bnD.4. A) Mutational antigenic profiling of bnD.8 and bnD.9 resistance. Line plots depicting positive (black) and negative (orange) site differential selection along the Env ectodomain for bnD.4 (4 µM) and bnD.9 (3 µM) in comparison. Mean differential selection of two independently generated and selected BF520 mutant libraries are shown. B) Sequence logoplots depicting site differential selection in regions of interest (C1, V3 and HR1) for bnD.4 and bnD.9. Height of letters indicates the excess fraction surviving for each mutation. C) Comparison of site differential selection between bnD.4 and bnD.9 for each amino acid position in Env. Sites that show a site differential selection >5.5 for both DARPins are shown in orange and numbered. D) Zoomed logoplots depicting mutational differential selection for sites which show escape both for bnD.4 and bnD.9. Source data

Several of these highly enriched mutations have been previously described to affect trimer conformation and are relevant for the stabilization of recombinant, soluble Env trimers. The C1 mutations E64K and H66R reduce spontaneous sampling of the CD4i conformation and are frequently used as stabilizing mutations in trimer design^15^. Likewise, substitutions in HR1 are used to generate closed Env immunogens, V570H and R585H increase the melting temperature of Env trimers, and K588Q stabilizes the interface^41^. The observed escape profile proved to be characteristic for V3-CD4i bnDs. bnD.4 displayed an escape profile centering on Y318 in V3 with only a few sites (residues 200, 213, 255, 388 and 560) outside V3 overlapping with V3-CD4i bnDs in the resistance selection (Extended Data Fig. 10c,d), indicating an effect of these mutations on V3 exposure. Most notably, in line with the V3 crown-specific bnD.4 depending less on trimer opening (Fig. 1a), we observed for bnD.4 less pronounced escape selection in trimer stabilizing regions.

Probing resistance conferring sites identified by mutational scanning (Fig. 6a) in the context of BF520 pseudovirus surprisingly showed increased sensitivity (Fig. 6d). C1 and HR1 mutants (E64N, H66Q, Q82L, E560G, A561T, K588Y and Q591A) displayed increased susceptibility to bnD.8 and bnD.9 (Fig. 6d). The same C1 and HR1 mutations also influenced sensitivity to bnAbs, with 10E8 and VRC01 largely increasing sensitivity, whereas VRC26.09 and tetrameric CD4-IgG2 showed a differential pattern with partial increasing and decreasing sensitivity (Fig. 6e). Trimer stabilization can result in slower entry kinetics, providing an extended window of action for inhibitors. We thus reasoned that this window may be optimally harnessed in the conventional pseudovirus neutralization test owing to the continuous presence of inhibitors, but not in the mutational scanning assay where inhibitors are removed after 3 h. Indeed, removal of the inhibitors after 3 h in the pseudovirus assay reverted the inhibition profile, rendering most viruses with stabilizing mutations more resistant against bnD.8 and bnD.9 (Fig. 6f). We conclude that trimer-stabilizing mutations reduce the ability of V3-CD4i bnDs to bind in the early docking phase of the entry process. However, this escape is not complete; the binding site of V3-CD4i bnDs is later exposed and can be efficiently exploited owing to the slowed entry kinetics of these mutants, explaining why trimer-stabilizing mutations did not emerge during the escape culture. Overall, the escape profile of the V3-CD4i bnDs demonstrates how important it is for HIV-1 to maintain a balance between trimer closing and rapid entry kinetics in order to optimize its defense against neutralization, and how difficult it is for the virus to occlude target sites without making global changes in Env functionality that can trigger vulnerabilities elsewhere on the spike.

Next, we considered whether αV3C-targeting bnDs themselves interfere with trimer stabilization and convey their neutralization activity through inducing gp120 shedding. This is conceivable because the interaction of V3 with CCR5 has been implicated in gp120 shedding during entry^45^. Furthermore, the postattachment active MPER antibodies 2F5 and 4E10 neutralize via irreversible shedding of gp120 (ref. ^35^). Considering that CD4 is a potent gp120 shedder itself, we chose a setup without CD4 triggering to explore the shedding capacity of bnD.8 and bnD.9. For this, we used a setup with prolonged (16 h) incubation of bnDs and JR-FL pseudotype virus, which we previously showed also allows MPER bnAb 2F5 to efficiently bind virions and induce shedding^45^. Consistent with the low binding capacity of bnD.8 and bnD.9 to untriggered trimer, we observed only a low to intermediate shedding capacity for several bnD.8 and bnD.9 variants (Fig. 6g). In contrast, the V3 crown-reactive bnD.3 (ref. ^20^) and the MPER bnAb 2F5 showed 92% and 82% shedding, respectively. Collectively, these results are consistent with a neutralization activity of bnD.8 and bnD.9 primarily directed against the CD4-triggered Env trimer. Although our results rule out substantial shedding induction before CD4 binding, more extensive premature shedding on partially CD4-triggered Env^46^ remains possible and warrants further investigation.

## Discussion

In this study, we defined an intermediate conformational state of the V3 loop that serves as a target for broad neutralization using DARPin technology (see Supplementary Discussion). The identification of bnD inhibitors targeting V3 in an open CD4-induced Env state (V3-CD4i bnDs) led to the definition of a previously unrecognized conformation of V3, a four-turn α-helix, which we term ‘αV3C.’ The discovery of the αV3C-helix illustrates the high conformational diversity of the Env trimer, where flexible subdomains adopt different conformations within the main open and closed states, providing conserved surfaces for recognition. Collectively, the identification of the αV3C-helix and the mechanisms involved in creating susceptibility to neutralization after CD4 binding create a deeper understanding of Env conformational transitions on which to build. Our findings mark αV3C as an additional target for effective entry therapeutics, prevention and vaccines to solicit the full potential of V3 inhibition. Together with the recently identified pan-neutralizing coronavirus antibodies that also exert their effect by binding a conserved α-helix exposed at a late entry stage^47,48^, αV3C-targeting bnDs demonstrate the potential of postattachment inhibition, calling for a systematic exploitation of this critical entry stage across viruses.

## Methods

### HIV-1 Env antibodies

HIV-1 Env-directed antibodies used in this study are listed in Supplementary Table 4.

### Peptides and mimetics

Peptides and mimetics were synthesized by Pepscan Presto. This included CD4M47 (ref. ^53^), linear V3 peptides, V3 mimetics and V3 peptides used for crystallization (V3 (BF520) [RKSVHLGPGQAFYATDGIIGEIR] and V3 (BG505) [NTRKSIRIGPGQAFYATGDIIGDI]).

Structure-arrested V3 crown mimetic peptides of strains MN and BG505.W6M.ENV.C2 (ref. ^54^) were designed to build anti-parallel β-strands that differ in the formation of interstrand hydrogen bonds (mimetic registers).

The following V3 peptides were used (sequences are indicated in square brackets): linear V3 peptides, linear 300-324 V3 (MN) [GGGGYNKRKRIHIGPGRAFYTTKNIIG] and linear 297-330 V3 (BG505) [TRPNNNTRKSIRIGPGQAFYATGDIIGDIRQAH]; structural V3 crown mimetics, V3-IY (MN) [KRIHIGPGRAFYTT^D^PP] and V3-IY (BG505) [KSIRIGPGQAFYAT^D^PP].

Biotinylated peptides were used for the peptide ELISA. The linear V3 (MN) peptide was biotinylated directly at the N terminus, cyclic V3-IY (MN) has a PEG08 linker between the peptide chain and biotin, and cyclic V3-IY (BG505) mimetics have a PEG04 linker between the peptide chain and biotin (see ref. ^54^). All synthetic peptides were ≥95% pure by analytical high-performance liquid chromatography (HPLC) and displayed electrospray mass spectrometry (MS) spectra consistent with the expected masses.

### Cell lines

293T cells (American Type Culture Collection) and TZM-bl cells (NIH AIDS Reagent Program) were cultivated in DMEM, high glucose, pyruvate supplemented with 10% heat-inactivated FBS, 100 U ml^−1^ penicillin and 100 µg ml^−1^ streptomycin (all from Gibco, Thermo Fisher Scientific) at 37 °C, 5% CO_2_ and 80% relative humidity. HEK 293T FreeStyle suspension cells (Thermo Fisher, 293F and Expi293F) for protein expression were maintained in serum-free FreeStyle 293F and Expi293F expression media (Thermo Fisher), respectively, according to the manufacturer’s instructions. A3.01-CCR5 (generated by our group)^55^ and Sup.T1-CCR5 cells (provided by J. D. Bloom and originally obtained from the NIH AIDS Reagent Program^56^) were maintained in RPMI with 10% heat-inactivated FCS, 100 U ml^−1^ penicillin and 100 µg ml^−1^ streptomycin. Cells were regularly tested for mycoplasma contamination and tested negative. No cell line authentication was performed.

### Env protein expression, purification and modification

Codon-optimized sequences of strain JR-FL gp120 wild-type and the V3 and V1V2 loop deletion mutants^57,58^ were custom synthesized (GeneArt), fused to a C-terminal AviTag and cloned into the CMV/R expression vector^59^. BG505.SOSIP.664 source plasmid was kindly provided by J.P. Moore and R. Sanders (Cornell University). The BG505.SOSIP.664 coding sequence was cloned into expression vector CMV/R with a C-terminal AviTag for in vitro biotinylation. The V1V2-deleted gp120 and SOSIP.664 constructs were generated as previously described^1^. BG505.DS.SOSIP.664 with swapped V1V2 from strains CAP256SU, c1080_c03 and WITO4160cl33 were custom synthesized (GeneArt) based on a previously described design^60^. Env proteins were produced in HEK 293T FreeStyle suspension cells. Env variants were expressed by transient transfection, and trimeric Env was expressed using a furin-expressing helper plasmid at a 3:1 ratio. HIV-1 Env proteins were purified from culture supernatants using *Galanthus nivalis* lectin resin (Vector Laboratories)^61^ and monobiotinylated using the BirA enzyme (Avidity). Superdex 200 size exclusion chromatography (SEC) (GE Healthcare) was used to derive the desired protein fraction (monomer or trimer). Trimer preparations were not further polished to remove partially opened trimers, allowing both fully shielded and partially V3-exposing trimers to enter DARPin selections. The two-domain sCD4 protein was expressed in *Escherichia coli* and purified as described^33^. The BG505.SOSIP.v4 (ref. ^15^) protein was a kind gift from M. van Gils and R. Sanders (Academic Medical Center, University of Amsterdam).

### DARPin selection by ribosome display

The principal methods of ribosome display, DARPin library design and DARPin selection by ribosome display have been described in detail^25,26,62–66^. Ribosome display is an in vitro translation method, where genetic information (that is, mRNA) and translated protein remain linked in a ternary complex, which in this case consists of the mRNA, the ribosome and the nascent DARPin, allowing the selection of DARPins of interest based on their binding properties and simultaneous derivation of their coding sequence. DARPin library screening in combination with ribosome display has been successfully used in diverse settings^26,63,67^. In this study, selection of Env target-specific DARPins was performed by ribosome display using an N3C DARPin library encoding DARPins with one N-capping, one C-capping and three internal ankyrin repeats^20,62,63,65,67,68^.

The panning targets to amplify Env-specific DARPins used in the five different selection campaigns (I–V) are summarized in Supplementary Fig. 1. Biotinylated targets were coupled to magnetic microbeads (Dynabeads MyOne T1, Invitrogen) via streptavidin, and the panning of ternary DARPin–mRNA–ribosome complexes was performed in 1.5-ml microtubes on a KingFisher Flex magnetic particle processor (Thermo Fisher Scientific). Five (selection I) or four (selections II–V) rounds of ribosome display were carried out, with decreasing immobilized target concentrations in the first rounds (250 nM (only selection A), 125 nM, 50 nM and 5 nM). In the round with lowest target concentration (5 nM), ternary complexes with the target were formed for 1 h followed by an off-rate step by adding a large molar excess (500 nM) of soluble target and incubating for another hour to select for high-affinity DARPins. In the last round (referred to as the rescue round), the immobilized target concentration was increased (50 nM) to amplify the remaining high-affinity DARPins. A pre-panning round for negative selection using BG505.SOSIP.664_ΔV1V2 or monomeric BG505.SOSIP.664 was applied for selections II–IV and V, respectively.

### DARPin expression and purification

DARPin pools retrieved from selection campaigns were cloned into pQE30-based expression vectors featuring an N-terminal His-tag and a C-terminal FLAG-tag. *Escherichia coli* XL1 blue bacteria were transformed with pools. A total of 190 individual clones were picked per selection. Small-scale cultures (1 ml) in LB medium were produced in deep-well plates for the initial binding and neutralization screens. When the bacterial culture reached an optical density at 600 nm (OD_600_) of 0.8, protein expression was induced with 0.5 mM isopropyl β-D-1-thiogalactopyranoside (IPTG; Sigma-Aldrich) for 4 h. To retrieve DARPins, bacterial pellets were lysed in 50 μl of B-PER II (Thermo Fisher Scientific) by incubation of the deep-well plate for 15 min at 1,300 r.p.m. and then for 50 min at room temperature (RT) without shaking. One milliliter of TBS/0.1% Tween/500 mM NaCl/0.1% BSA (pH 8) was added, and the equilibrated lysate was centrifuged (3,000 *×* *g*, 20 min, 4 °C) to remove cell debris. Finally, 900 µl of the supernatant was transferred to a new 96-well deep-well plate, and this crude extract was stored at −20 °C. DARPins were purified from 900 µl of bacterial crude extract in 96-well plates using HisPur Cobalt Spin Plates (Thermo Scientific). Cytotoxic imidazole (Sigma-Aldrich) was removed from DARPin eluates by extensive washing with PBS on AcroPrep 96-well filter plates (3 kDa molecular weight cutoff (MWCO)).

DARPins from large-scale batches (0.2–1 l) were purified by Ni-NTA affinity chromatography and by SEC^69^. SEC was performed on an ÄKTA Purifier system (GE Healthcare) with a Superdex 200 10/300 GL column (GE Healthcare) and PBS as running buffer. Monomeric fractions were concentrated (Amicon Ultra Centrifugal Filters, Merck Millipore) and stored at −20 °C.

### bnD Fc constructs

Indicated bnDs were also expressed as bivalent bnD crystallizable fragment (Fc) fusions. To this end, the DARPin sequence was fused to the N terminus of the coding sequence for the Fc region of the human IgG1 heavy chain (IGHG1, GenBank NC_000014.9, REGION: 105741473.105743070), preserving the hinge region, and cloned into a pcDNA3.1 expression vector. bnD Fcs were expressed by transient transfection in 293F or Expi293F cells and purified from the supernatant on a protein G affinity chromatography column equilibrated in 20 mM phosphate buffer, pH 7.0, and eluted with 100 mM glycine, pH 2.7. The eluate was adjusted to pH 4.0 using 1 M Tris buffer, pH 8.7, concentrated using an Amicon filter unit (10,000 kDa MWCO, Millipore) and immediately purified by SEC on a Superdex 200 10/300 GL Increase column (GE Healthcare) equilibrated in PBS. Monomeric fractions were concentrated and sterile-filtered before use in neutralization assays.

In an alternate approach, bnD–antibody complexes were generated to mimic bnD-like antibody molecules making use of the DARPins’ N-terminal His-tag. To this end, 6x-His-Tag monoclonal antibody (HIS.H8; Invitrogen, MA1-21315-1MG) was incubated undiluted with 3-fold molar excess of purified bnD for 1 h at RT and purified by SEC on a Superdex 200 10/300 GL Increase column equilibrated in PBS. Monomeric fractions were concentrated and sterile-filtered before use in neutralization assays.

### Detection of DARPin and mAb binding to target proteins by ELISA

White high-binding 384-well microplates (Corning) were coated with 66 nM NeutrAvidin (Thermo Fisher Scientific) overnight at 4 °C or for 1 h at RT and blocked with TBS supplemented with 3% BSA for 1 h at RT. During the 1 h at RT, 20 nM monobiotinylated target was immobilized to the plates. Target Env proteins were either probed unliganded or triggered with 50 nM (BG505.SOSIP.664) or 100 nM sCD4 (BG505.SOSIP.664.V4.1). After this and all following incubation steps, three washes with TBST (TBS containing 0.1% Tween 20 (Sigma-Aldrich), pH 7.5) were performed. Serial dilutions of purified DARPins or mAbs were added in TBSTB (TBST with 0.5% BSA (Sigma-Aldrich), pH 7.5). Unbound material was washed off in TBST, and bound DARPins were detected via their FLAG-tag using 1:15,000 diluted mouse anti-FLAG antibody (clone M2, Sigma-Aldrich, F1804 and F3165). After incubation with 1:15,000 diluted polyclonal alkaline phosphatase conjugated anti-mouse IgG secondary antibody (Sigma-Aldrich, A3562), a chemiluminescent substrate (Tropix CDP-Star, Thermo Fisher Scientific) was added. Emission of relative light units was recorded on a PerkinElmer EnVision Multilabel Reader. Binding of mAbs was detected with 1:15,000 diluted polyclonal anti-human IgG (Fc-specific) alkaline phosphatase conjugated antibody produced in goat (Sigma-Aldrich, I2136).

### Env pseudoviruses

Env-pseudotyped viruses were prepared by co-transfection of HEK 293T cells with plasmids encoding the respective *env* genes and the luciferase reporter HIV vector pNLluc-AM as described^70^. A full list of Env-pseudotyped viruses generated with corresponding GenBank entry and subtype is provided in Supplementary Table 1. The plasmid collection of JR-CSF Env alanine mutants^6,71,72^ was kindly provided by D. Burton (The Scripps Research Institute).

### Neutralization assay using Env-pseudotyped virus

The neutralization activity of DARPins and mAbs was evaluated on TZM-bl cells using Env-pseudotyped viruses in a 384-well format^70^. Input of Env pseudoviruses was chosen to yield virus infectivity corresponding to 5,000–20,000 relative light units (RLU) in the absence of inhibitors as measured on a Dynex MLX. Readouts of 384-well plates were done on the PerkinElmer EnVision Multilabel Reader. The DARPin or antibody concentrations causing 50% reduction in viral infectivity (half-maximum inhibitory concentration, IC_50_) were calculated by fitting data to sigmoid dose–response curves (variable slope) using Prism (GraphPad Software). If 50% inhibition was not achieved at the highest or lowest inhibitor concentration, a greater than or less than value was recorded.

Primary neutralization screen: As DARPin yield from 1 ml *E. coli* cultures is limited, the virus neutralization screen of DARPin pools was restricted to a single replicate per virus using DARPin preparations from small-scale purifications on HisPur Cobalt Spin Plates (see above) diluted 1:6 in TZM-bl cell culture medium. To control for unspecific effects, all DARPin clones were tested for activity against murine leukemia pseudovirus (MuLV). Viruses probed in these initial neutralization screens were CAP45_2_00_G3, C1080_c03, BG505_W6M_ENV_A5_T332N, WITO4160 clone 33 and JR-FL to cover clades and targets used in the panning. To compare potency of DARPins, we calculated a neutralization score. Individual inhibitory activity of a DARPin against a specific virus in the 5-virus panel in the range of 50 ≤ 70, >70 ≥ 90 or >90% neutralization received a score of 1, 2 or 3, respectively. These scores were multiplied by the sum of the number of strains neutralized >50% to calculate overall neutralization score, with a maximum of 75. DARPins with a neutralization score below 5 were considered non-neutralizing. DARPins with a neutralization score of 5–14 were categorized as weakly neutralizing DARPins, and DARPins with a score of ≥15 were categorized as broadly neutralizing.

### Preattachment and postattachment activity assay

The inhibitory capacities of inhibitors at preattachment and postattachment states were analyzed as previously described on A3.01-CCR5 target cells using NLlucAM reporter virus^73^. In this assay, inhibitor treatment is initiated either before or after attachment of the virus to the cells. bnAbs PG128, VRC01 and 10E8 were used as controls. In agreement with their known capacity to bind prefusion closed Env, bnAbs PG128 and VRC01 show higher preattachment but lower postattachment neutralization activity, whereas MPER bnAb 10E8 shows profound postattachment activity. The assay measures three activities: total inhibitory activity, preattachment activity and postattachment activity. Total activity corresponds to the traditional neutralization assay in which the virus is pretreated with the inhibitor and the inhibitor remains present throughout. Preattachment and postattachment neutralization activity are assessed in specifically tailored setups in the same assay. Inhibitors are tested at a single dose in all three setups. The total activity is set to 100%, and the relative contribution of preattachment and postattachment neutralization activity is expressed in relation to it.

NLlucAM reporter virus stocks were adjusted to yield a firefly luciferase activity of approximately 10,000 RLU per 96 wells in absence of inhibitors.

To test total inhibitory activity (which covers both the preattachment and postattachment stages), virus was preincubated with a single dilution of bnD or bnAb for 1 h at 37 °C. The concentrations used were 1 µM (bnDs) and 6.4 nM (bnAbs). The pretreated virus was then spinoculated at 23 °C onto 1 × 10^5^ A3.01-CCR5 target cells in RPMI with 50 mM HEPES and 10 μg ml^−1^ DEAE per well for 2 h at 1,200 G. Unbound virus and inhibitors remained with the cells during the subsequent cultivation at 37 °C.

To test preattachment neutralization activity, we altered the setup such that after spinoculation at 23 °C, unbound virus and inhibitors are washed off twice with culture medium and centrifugation at 450 × *g* for 2 min. This condition provides information on how much of the binding and neutralization can occur before finalization of CD4 engagement and whether the affinity of the binding is high enough to sustain washing. Thus, inhibitory capacities measured after washout of inhibitors reflects neutralization that was initiated prior to receptor engagement. To test postattachment inhibition, we spinoculated virus without inhibitors onto A3.01-CCR5 cells. Inhibitors were added following spinoculation at 23 °C before raising the temperature to 37 °C.

In all three setups, cells were incubated for 65 h at 37 °C, and infectivity was determined by firefly luciferase production from the lysed cells. Total activity in samples was set to 100% inhibition, and postattachment inhibition was expressed relative to this value. Preattachment activity was expressed in relation to the total inhibitory activity, which was set to 100%.

### Detection of gp120 shedding from bead-immobilized virus

The capacity of the MPER bnAb 2F5 and DARPins to induce gp120 shedding from virions was assessed as previously described^45^. In brief, JR-FL Env pseudotype virus was generated in 293T cells transfected with mouse CD4. bnAb and bnD concentrations were adjusted to 20-fold above the respective neutralization IC_50_ of the inhibitors against JR-FL. Virus preparations (adjusted to 7 × 10^6^ RLU per reaction, as measured by pseudovirus infectivity of TZM-bl cells) were incubated with inhibitors for 16 h at 37 °C. Virus particles were captured with magnetic Dynabeads coated with rat anti-mouse CD4 mAb L3T4 (Thermo Fisher Scientific) and shed gp120 were removed by two washes in TBS + 2% BSA. The bead-trapped pseudovirus was then lysed in TBS + 1% Empigen, and gp120 and p24 content of the virus lysate was measured by ELISA as previously described^74^. In brief, virus preparations were dissolved in 1% Empigen (Fluka Analytical) and probed for gp120 and p24. Gp120 was captured on anti-gp120 D7324-coated (Aalto Bio Reagents) immunosorbent plates and detected with biotinylated mAb 2G12 (Polymun) and streptavidin-coupled alkaline phosphatase (GE Healthcare). P24 was captured on anti-gp120 D7320-coated (Aalto Bio Reagents) plates and detected using alkaline phosphatase-coupled antibody BC1071-AP (Aalto Bio Reagents).

To calculate the percentage of shedding, for each probed condition, the gp120 content was normalized to the p24 content and shedding was displayed as the percent reduction of virion-associated gp120 content compared with mock-treated controls.

### Sequence analysis of DARPins and Env clones

Sequencing of DARPin and *env* genes was performed at Microsynth AG.

### DARPin binding to cell surface-expressed Env

DARPin binding to Env expressed on HEK 293T cells was detected as previously described^33^. In brief, HEK 293T cells were co-transfected with the desired Env expression plasmid and the pCMV-rev expression helper plasmid in a 4:1 ratio for 36 h. Cells were then incubated with DARPins in the presence or absence of two-domain sCD4 for 20 min at RT. Detection was performed via the FLAG-tag using PE-conjugated anti-FLAG L5 antibody (BioLegend, 637309) on a FACSVerse system (BD Biosciences) and analyzed using FlowJo software (version 10; FlowJo, LLC).

### Crystallization and protein structure determination

For crystallization, DARPins bnD.8 and bnD.9 were subcloned into plasmid pQlq_H10_3C containing a human rhinovirus 3C protease-removable 10x histidine tag. DARPins were expressed and Ni-NTA purified as described above. His-tags on DARPins were removed by human rhinovirus 3C protease (Sigma-Aldrich) (2% w/w) overnight while dialyzing against PBS. Uncleaved DARPins and 3C protease were removed by reverse Ni-NTA chromatography. Monomeric DARPin fractions were isolated by SEC on an ÄKTA Pure system (GE Healthcare) with HiLoad 16/600 Superdex 75 pg or Superdex 200 10/300 GL columns (GE Healthcare) and 10 mM HEPES pH 7.4, 150 mM NaCl as running buffer. Monomeric DARPin fractions were supplemented with 2-fold molar excess of V3 peptide, then the complexed protein was separated by size exclusion using a HiLoad 16/600 Superdex 75 pg and 10 mM HEPES pH 7.4, 150 mM NaCl as running buffer. Protein complexes were concentrated (Amicon Ultra Centrifugal Filters, Merck Millipore) to 20 mg ml^−1^.

Sparse-matrix screens (Hampton Research, Molecular Dimensions and Qiagen) in a sitting-drop vapor diffusion format at 20 °C were set up to identify initial crystallization conditions; focus screens with pH and precipitant gradients were used to refine initial conditions. Crystals were flash-frozen (liquid N_2_) in mother liquor supplemented with 40% ethylene glycol. Data were collected on beamlines X06DA and X06SA at the Swiss Light Source (Paul Scherrer Institute) at a wavelength of 1.0 Å using an Eiger detector system (Dectris Ltd).

Data were processed using XDS (version Jan 31, 2020)^75^, Aimless (version 0.7.4)^76^ and autoPROC (version 2.3.13)^77^ with 5% of data set aside for calculating the *R*_free_ value. Initial phases were obtained by molecular replacement using Phaser (version 2.8.3)^78^ with the structure of the full consensus N3C (PDB 2QYJ) as a search model. Refinement was done using REFMAC5 (version 5.8.0267)^79^ and phenix.refine (version 1.19_4080)^80,81^, followed by model building in Coot (version 0.9.3)^82^. All V3 peptides could be completely built into difference electron density during refinement. Analysis of binding interfaces was done with LigPlot+ (version 2.2.5)^83^. Figures were prepared with PyMOL (version 2.1; Schrödinger, LLC).

### Cryo-EM sample preparation

BG505.SOSIP.664 and CD4 D1D2 domains were expressed and purified as described^84,85^. 8x-His-tagged bnD.9 was expressed by transiently transfecting Expi293F cells (Thermo Fisher Scientific) and purified using Ni-NTA agarose (cOmplete His-Tag Purification Resin, Roche). To make a ternary complex of BG505.SOSIP.664–CD4 D1D2– bnD.9, the purified BG505.SOSIP.664 was incubated with a 3-fold molar excess of CD4 D1D2, followed by incubation with a 3-fold molar excess of purified bnD.9. The complex was then purified by SEC using a HiLoad 16/600 Superdex 200 pg column (GE Healthcare) with PBS as running buffer. We deposited 2.3 µl of the complex at a concentration of 1 mg ml^−1^ onto a C-flat grid (https://www.protochips.com). The grid was vitrified using an FEI Vitrobot Mark IV with a wait time of 30 s, blot time of 3 s and blot force of 1.

### Cryo-EM data collection, processing and structure refinement

Cryo-EM data for BG505.SOSIP.664 in complex with bnD.9 and sCD4 (D1-D2) were collected using the Leginon software^86^ installed on a Titan Krios electron microscope operating at 300 kV, equipped with a Gatan K3-BioQuantum direct detection device. The total dose was fractionated for 3 s over 60 raw frames. Motion correction, contrast transfer function (CTF) estimation, particle extraction, two-dimensional (2D) classification, ab initio model generation and three-dimensional (3D) refinements were carried out in cryoSPARC (version 2.15)^87^. The final 3D reconstruction was obtained using nonuniform refinement with C1 symmetry. The interface region between bnD.9 and BG505.SOSIP.664 was locally refined by using a mask that included the whole bnD.9 molecule and the interacting region of the gp120 subunit. The density for BG505.SOSIP.664 and CD4 (D1-D2) was modeled using Protein Data Bank (PDB) entry 5VN3 (ref. ^23^) as the initial template; the density for bnD.9 and the V3 region of gp120 was modeled using the crystal structure of bnD.9–V3 (BF520) peptide reported in this manuscript as the initial template.

Automated and manual model building were iteratively performed using real-space refinement in Phenix (version 1.19)^88^ and Coot (version 0.9.4)^82^, respectively. Local resolution was estimated using ResMap (version 1.1.4)^89^. Half maps were provided to the Resolve Cryo-EM tool in Phenix to support manual model building. Geometry validation and structure quality assessment were performed using EMRinger^90^ and MolProbity^91^ both implemented in Phenix (version 1.19). Map-fitting cross-correlation (Fit-in-Map tool) and figure preparation were carried out using PyMOL (version 2.4.2) and UCSF Chimera (version 1.15)^92^. A summary of the cryo-EM data collection, reconstruction and refinement statistics is shown in Table 1.

### Molecular dynamics

A molecular dynamics simulation was carried out in order to characterize the stability of αV3C within the V3 loop on the fully glycosylated BG505.SOSIP.664 protomer under near-physiological conditions. One of the protomers from the cryo-EM structure (gp41/120 and CD4) was extracted. N-linked mannose-5 glycans were modeled onto each sequon using the Python package Glycosylator (version 1.0)^93^. The structure was then solvated in a 17 Å padding water box and neutralized by the addition of NaCl salt at a concentration of 150 mM.

The simulation was performed with the ACEMD molecular dynamics engine and the CHARMM36 force field. TIP3P water parameterization was used to describe the water molecules. The periodic electrostatic interactions were computed using particle-mesh Ewald (PME) summation and a grid spacing smaller than 1 Å. A constant temperature of 310 K was imposed with Langevin dynamics, and constant pressure of 1 atm was maintained with a Berendsen barostat. During equilibration, the backbone atoms were restrained with harmonic restraints. The system was first minimized by 5,000 conjugate gradient steps and then equilibrated for 20 ns before removing all restraints. The hydrogen mass repartitioning scheme was then used to achieve 4-fs time steps. The unrestrained molecular dynamics simulation was performed up to 2 μs.

### Molecular modeling of the IgG engaging with the HIV-1 Env–CD4 complex

The HIV-1 CD4 complex was modeled by combining the cryo-EM structure of BG505.SOSIP.664–sCD4–bnD.9 with the full-length prediction of CD4 receptor from the AlphaFold Protein Structure Database^94^. The CD4 transmembrane regions were then embedded into a 1-palmitoyl-2-oleoyl-sn-glycero-3-phosphocholine (POPC) lipidic bilayer mimicking the T-cell plasma membrane. A molecular dynamics simulation combining an elastic network with the Martini 2.0 force field^95^ was prepared with CHARMM-GUI^96^. The system was simulated with GROMACS^97^ for up to 1 µs. The last frame was considered to be a starting point for the modeling of the IgG interaction.

A possible approach angle of the antibody was determined with the crystal structure of a Fab engaging with a partially helical V3 (PDB 5V6L). The helical peptide was first superimposed on the corresponding region of the αV3C. The position of the Fab was then used to superimpose the full-length IgG antibody (PDB 1HZH). This process was repeated for each protomer of the HIV-1 Env, and the complex with the lowest clashes was saved.

### Protein–protein docking

The RosettaDock procedure was used for protein–protein docking^98^. For each structure, 700 poses were generated. The structures with the lowest energy and a root mean squared deviation (r.m.s.d.) to the initial pose smaller than 5 Å were considered as successful docking.

### Rosetta design

The Rosetta protein design suite was used to remodel and improve the interface between bnD.9 and gp120. The initial structure of bnD.9 in complex with gp120 was first relaxed with the FastRelax protocol (10 structures). The structure with the lowest Rosetta score was then chosen for the design of the bnD9–gp120 interface using the FastDesign protocol (300 structures). Finally, the structure with the lowest Rosetta score was selected for experimental validation.

### Env sequence entropy assessment

For each residue position, Shannon’s entropy was calculated based on the aligned sequences (either 42-virus panel or 7,590 HIV-1 group M Env sequences downloaded from the Los Alamos National Laboratory database) with the formula

$${\mathrm{Entropy}}=-\mathop{\sum }\limits_{i=1}^{20}p\left({x}_{i}\right){{\log }}\left[p\left({x}_{i}\right)\right]-{p}_{\mathrm{gap}}{{\log }}(1/n)$$

in which *x*_*i*_ is a standard amino acid, *p*(*x*_*i*_) is the frequency of amino acid *x*_*i*_ at this position, and *p*_gap_ is the frequence of gap in this position. The entropy is normalized by dividing it by the maximum value of entropy, so that normalized entropy values are between 0 and 1.

### Env mutational antigenic profiling

For Env mutational antigenic profiling using BF520 Env libraries^38,99,100^, 5 × 10^5^ infectious units of two independently generated BF520 mutant virus libraries were neutralized with bnD.8, bnD.9 and bnD.4 at an estimated IC_99_ concentration (0.5 µM, 1 µM and 1 µM of DARPin, respectively) for 1 h. Neutralized libraries were infected into 1 × 10^6^ SupT1.CCR5 cells in R10 (RPMI supplemented with 10% FBS, 10 mM HEPES, 100 U ml^−1^ penicillin and 100 μg ml^−1^ streptomycin), in the presence of 100 µg ml^−1^ DEAE-dextran. At 3 h after infection, cells were spun down and resuspended in 1 ml of fresh R10 without DEAE-dextran. At 12 h after infection, cells were washed with PBS, and nonintegrated viral complementary DNA was isolated. In parallel to each DARPin selection, each mutant virus library was also subjected to a mock selection. Additionally, four 10-fold serial dilutions of each mutant virus library were used to infect 1 × 10^6^ cells to serve as an infectivity standard curve. Typically, we observed a remaining infectivity of 1–5% compared with unselected library. Selected and mock-selected viral cDNA was then sequenced with a barcoded subamplicon sequencing approach as previously described^100^. Primers used are described in ref. ^99^, Data S6. This approach involves introducing unique molecular identifiers (UMIs) during the Illumina library preparation in order to further reduce the sequencing error rate. The mean differential selection at each site was calculated using dms_tools2 (version 2.6.6) from codon counts from two independent experiments as previously described^100^.

### In vitro culture-based escape selection

Healthy donor PBMCs were isolated from buffy coats from anonymous blood donations from healthy individuals obtained by the Zurich Blood Transfusion Service (https://www.zhbsd.ch) under a protocol approved by the local ethics committee. PBMCs were stimulated and CD8 was depleted as described^101^ and cultivated in RPMI with 10% heat-inactivated FCS, 1% penicillin/streptomycin and 100 U ml^−1^ human recombinant interleukin-2 (IL-2) (Hoffmann-La Roche). To test if and how HIV-1 evades pressure by bnD.8 over multiple rounds of infection, 5 × 10^6^ CD8-depleted PBMCs were infected with HIV at a multiplicity of infection (MOI) of 0.1 in two parallel experiments, one with the clonal, replication-competent BF520 wild-type virus and the other using the BF520 mutational virus library virus stock that contains Env mutant viruses that may aid the escape selection. Selections were started at three concentrations of bnD.8 covering its IC_80_ against BF520 (30 nM, 60 nM and 90 nM) in infection cultures totaling 2 ml. In parallel, control cultures were maintained that were infected with BF520 wild-type and BF520 library in the absence of DARPin (mock culture). On days 7 and 14 after infection, virus replication was monitored by titrating culture supernatant on TZM-bl cells. In each passage, once virus replication in bnD-treated culture was established, cell-free culture supernatant was collected and used to infect a new batch of PBMCs. bnD.8 doses were gradually increased over 9 weeks of culture and reached 3 μM in the last passage. Viral supernatants were probed for sensitivity to bnD.8 in TZM-bl neutralization assays to determine IC_50_ values of DARPins against the virus population.

To confirm resistance on a clonal level, viral RNA was isolated from the supernatant in the last passage using the Qiaprep Viral RNA Kit (Qiagen) and reverse-transcribed using the PrimeScript One Step RT-PCR Kit (Takara). Envelope genes were amplified using KAPA HiFi DNA polymerase and cloned into the pcDNA3.1/V5-His (Invitrogen) vector using the In-Fusion protocol (Clontech) according to the manufacturer’s instructions. *Env* genes were sequenced, and plasmids encoding Env were used to generate pseudotyped viruses as described above.

### Programs

Figures were generated with programs for which the purchased software subscription includes journal publication: GraphPad Prism (version 9; GraphPad Software), R and BioRender.com (paid subscription; BioRender). Figures displaying structures were generated using PyMOL or ChimeraX. Figures were assembled and finalized in Affinity Designer (Serif Europe Ltd). Language editing was performed with DeepL Translate and DeepL Write.

### Reporting summary

Further information on research design is available in the Nature Portfolio Reporting Summary linked to this article.

## Online content

Any methods, additional references, Nature Portfolio reporting summaries, source data, extended data, supplementary information, acknowledgements, peer review information; details of author contributions and competing interests; and statements of data and code availability are available at 10.1038/s41594-023-01062-z.

## Supplementary information

Supplementary Information Supplementary Note, Discussion, Tables 1–4, Figs. 1–4, and References.

Reporting Summary

## Data Availability

The structural data on DARPin–V3 complexes generated in this study (Figs. 4 and 5 and Supplementary Fig. 4) have been deposited in the PDB under accession codes 7Z7C (bnD.8–V3 (BF520)) and 8AED (bnD.9–V3 (BG505)). The cryo-EM structure of bnD.9–BG505.SOSIP.664–sCD4 was deposited in the PDB under 7TXD and the Electron Microscopy Data Bank (EMDB) under EMD-26157. Tables 1 and 2 show corresponding data collection and refinement statistics. Other publicly available datasets from the PDB used in this study (Figs. 4 and 5) are accessible under 6MEO (CCR5–gp120–sCD4), 2B4C (X5–gp120), 2QAD (412D–gp120), 5VN3 (17b–BG505.SOSIP.664–sCD4), 5V6L (10A37–V3), 7B4U (bnD.2–V3), 7B4W (bnD.3–V3), 3MLV (447-52D–V3), 3GO1 (268-D–V3) and 5FUU (PGT151–JR-FL EnvΔCT). Source data for all other figures are provided as Supplementary Tables. Additional source data retrieved from ref. ^20^ can be found online at 10.1038/s41467-021-27075-0. HIV sequences downloaded from the Los Alamos National Laboratory database are accessible at https://hiv.lanl.gov. The AlphaFold model for CD4 can be accessed at https://alphafold.ebi.ac.uk/entry/P01730. Source data are provided with this paper.

## Source data

Source Data Fig. 1 Statistical source data.
Source Data Fig. 2 Statistical source data.
Source Data Fig. 3a Statistical source data.
Source Data Fig. 5 Statistical source data.
Source Data Fig. 6 Statistical source data.
Source Data Extended Data Fig. 1 Statistical source data.
Source Data Extended Data Fig. 8 Statistical source data.
Source Data Extended Data Fig. 9 Statistical source data.
Source Data Extended Data Fig. 10 Statistical source data.
