# Supplementary material for: Trapping the HIV-1 V3 loop in a helical conformation enables broad neutralization
Source: Nat Struct Mol Biol. 2023 Aug 21;30(9):1323–36. doi: 10.1038/s41594-023-01062-z (PMC10497408; doi:10.1038/s41594-023-01062-z)
Supplement: Supplementary file 1 — Supplementary Note, Discussion, Tables 1–4, Figs. 1–4, and References. [file 41594_2023_1062_MOESM1_ESM.pdf]

nature structural & molecular biology

Article https://doi.org/10.1038/s41594-023-01062-z

Trapping the HIV-1 V3 loop in a helical conformation enables broad neutralization

In the format provided by the authors and unedited

Supplementary information

Trapping the HIV-1 V3 Loop in a Helical Conformation Enables Broad Neutralization

Matthias Glögl[1], Nikolas Friedrich[1]§, Gabriele Cerutti[2]§, Thomas Lemmin[1]§, Young D. Kwon[3]§, Jason Gorman[3], Liridona Maliqi[1], Peer R. E. Mittl[4], Maria C. Hesselman[1], Daniel Schmidt[1], Jacqueline Weber[1], Caio Foulkes[1], Adam S. Dingens[5], Tatsiana Bylund[3], Adam S. Olia[3], Raffaello Verardi[3], Thomas Reinberg[4], Nicolas S. Baumann[1], Peter Rusert[1], Birgit Dreier[4], Lawrence Shapiro[2,6], Peter D. Kwong[3], Andreas Plückthun[4], Alexandra Trkola[1*]

1 Institute for Medical Virology, University of Zurich (UZH), Zurich, Switzerland.
2 Zuckerman Mind Brain Behavior Institute, Columbia University, New York, NY, USA
3 Vaccine Research Center, National Institute of Allergy and Infectious Diseases, National Institutes of Health, Bethesda, MD, USA.
4 Department of Biochemistry, University of Zurich (UZH), Zurich, Switzerland.
5 Fred Hutchinson Cancer Research Center, Seattle, WA, USA
6 Department of Biochemistry and Molecular Biophysics, Columbia University, New York, NY, USA
§ contributed equally
* correspondence: trkola.alexandra@virology.uzh.ch

Content

1) Supplementary note: DARPin N-cap displacement 2
2) Supplementary Discussion 4
3) Supplementary Tables 7
Table S1: Virus panels 7
Table S2: Neutralization breadth and potency of V3 directed DARPins and antibodies 8
Table S3: Neutralization of JRCSF variants 10
Table S4: Inhibitor Source 11
4) Supplementary Figures 12
S1: Flow cytometry and gating strategy 12
S2: Sequence entropy in V3 and the CD4 binding site 13
S3: V4-CD4i bnDs are differentially influenced by N-cap framework mutations or removal 14
S4: Conservation of V3 15
4) References 16

1) Supplementary note: DARPin N-cap displacement

Structural analysis of bnD.8 and bnD.9 (Fig. 4) pointed towards a (partial) unfolding of the N-cap or a displacement with N-cap residue D28 acting as a hinge. This displacement appears to be required for bnD.9 accessing the V3-stem at this angle in the open trimer structure due to space constraints (Fig. 4D), and it leads to an additional interface with the gp120 bridging sheet. A hydrogen bond forms between DARPin H49 and Env K420 in the gp120 bridging sheet. Of note, residue H49 is positioned in the inner domain of the DARPin (Extended Data Fig. 4B) and is not available for interaction when the cap is not displaced. We therefore investigated the N-cap displacement observed with bnD.9 further, as it may provide insights into the general accessibility of αV3C. Framework regions of N- and C-caps are conserved in the DARPin library design to maintain the functionality of the DARPin scaffold — mutations in these regions are therefore rare. We detected an unusual D15L mutation in the N-cap of bnD.9 that results in rearrangement by pushing R19 and subsequent loss of two N-cap-stabilizing salt bridges made by R19 (Supplementary Fig. 3a). Notably, we observed unusual mutations in the interface of N-cap and first internal ankyrin repeat in 5 of 8 V3-CD4i bnDs but not in any of the V3-crown bnDs (Extended Data Fig. 2). This strongly hints at a feature selected during the ribosome display selection, which allows mutagenesis to happen during PCR. The remaining V3-CD4i bnDs, including bnD.8, had the original scaffold sequence, suggesting that they may not require an N-cap shift to bind. To investigate this further, we performed docking analyses with bnD.8 using a series of frames derived from the MD simulations, showing that bnD.8 can successfully approach the target by a change in the angle of the protruding αV3C (Extended Data Fig. 7D). To explore the importance of the N-cap for binding and neutralization and the influences of potentially destabilizing mutations, we generated constructs without N-cap, and with non-mutated (D15, as in bnD.8) or mutated (D15L, as in bnD.9) N-cap. Removal of the N-cap increased the potency for bnD.9 substantially (11.8-fold, Supplementary Fig. 3B). Interestingly, restoring the N-cap also increased the potency of bnD.9 to a similar degree, and the median potency of bnD.8 without N-cap was only moderately better compared to bnD.8 (3.8-fold). Taken together, this indicates that the efficacy of bnDs with or without a mutated N-cap was similar overall. Conversely, however, mutation of the bnD.8 N-cap reduced potency. This pattern is consistent with a scenario in which binding for both DARPins is initiated with the N-cap in place, and N-cap displacement occurs secondary to this.

Although N-cap displacement does not appear to be required for neutralization, the truncation of both bnD.9 and bnD.8 likely benefits the interaction with the trimer by reducing overall steric hindrance and introducing an additional hydrophobic interface with the gp120 bridging. Thus we explored the potential for enhancing the bridging-sheet interactions by performing an in-silico optimization of bnD.9 with Rosetta [1,2] (Figure S8C). Mutations suggested to augment the binding energy (L43I, F47E, and I52M) indeed resulted in an increase in potency and breadth of truncated bnD.9 (Supplementary Fig.

3D; Extended Data Fig. 3). Within the 42-virus panel examined, only three strains were resistant (ZEnv32_0111_5, ZEnv16_1202_7, and Q769_h5), the latter two of which carry a deletion in V3 that is expected to twist the helix interface, further highlighting the importance of the αV3C helix for engagement by V3-CD4i bnDs.

To further investigate accessibility of αV3C for antibody-like molecules in vitro, we generated C-terminal Fc-fusion proteins composed of the most potent versions of bnD.8 and bnD.9, respectively, and the constant region of human IgG1. Although a substantial loss in neutralization capacity against Tier-2 viruses was evident, some neutralization was retained, and constructs stayed active against Tier-1 strain Bal26 and enhanced activity against SF162 (Extended Data Fig. 8B). Upon binding αV3C, the bnD C-terminus is expected to be close to the host cell membrane. We therefore considered that fusion of the Fc to the C-terminus may have added further access constraints. We therefore investigated an alternate antibody-like bnD complex in which the bnD is connected N-terminally to the antibody part. To achieve this, we complexed N-terminally his-tagged bnD with an anti-his-tag antibody. Although this antibody-bnD complex is larger than the bnD-Fc fusion proteins, we observed a higher neutralization capacity towards Tier-2 viruses (Extended Data Fig. 8B). Collectively, these data support the notion that access for αV3C antibodies is possible. In the context of MPER bnAbs, IgG3 molecules were considered advantageous for accessing the MPER region near the viral lipid membrane due to their extended and more flexible hinge region [3,4]. Interestingly, our model also suggests that αV3C, which is located close to the cell membrane in the CD4-bound state, would also be more accessible to IgG3 molecules.

2) Supplementary Discussion

In this study, we defined an intermediate conformational state of the V3 loop that serves as target for broad neutralization. High-resolution structural analysis of flexible proteins and subdomains such as the V3 loop on HIV-1 Env requires fixation, usually by high-affinity ligands. Therefore, to obtain information on native conformations, the selected ligands should ideally capture the protein without forcing an induced fit upon binding. Using the DARPin technology, we have developed a strategy to localize and resolve V3 conformations associated with broad neutralization capabilities. Due to their rigid binding surface, DARPins are often highly dependent on the conformation of the target, and, as we show here, can be used to define novel HIV-1 Env substructures that have eluded conventional structural analysis of antibody-ligated complexes.

To prove the concept that V3 is amenable to broad neutralization beyond the established V3-glycan bnAb epitopes, we generated a range of broadly neutralizing DARPin (bnD) inhibitors targeting V3 in an open CD4-induced Env state. The selected bnDs matched the breadth of the V3-glycan bnAbs (60-79%) but showed lower potency, which is likely due to their mode of action post CD4 binding [5]. Indeed, all post-CD4 attachment inhibitors, including antibodies that act after CD4 binding like MPER bnAbs, show lower potency compared with pre-attachment inhibitors [6-11].

Using the novel V3-CD4i bnDs to define the target site for broad neutralization, we show that the V3 adopts a previously unrecognized conformation, a 4-turn alpha helix that we term αV3C. We postulate that this conformation is also sampled without ligand binding during the entry process, based on several lines of evidence. We observed the αV3C conformation in complex with two unrelated DARPins obtained from independent Ribosome Display selections using different panning targets. Molecular dynamics simulations highlight that the helix is stable in the absence of the DARPin ligand and is supported by additional salt bridges between conserved residues that are not functionally required for co-receptor interaction. Considering the strong immune pressure on V3, it would be difficult to comprehend, that such an extended helix-forming stretch is maintained without functional relevance. In the five independent screens that gave rise to aV3C DARPins, a range of Env probes were included comprising open and closed Env conformations that all at least partially expose the V3 region. Whether a fraction of the panning proteins spontaneously sampled the aV3C conformation or whether binding to unstructured V3 sufficed for selection, cannot be deferred from these experiments but irrespective of this, these parallel selections underline that aV3C targeting molecules are not a chance finding and the appropriate target regions were sufficiently exposed. It is conceivable that the amphipathic αV3C helix interacts with the host membrane during coreceptor interaction through its hydrophobic face. Indeed, helix-breaking amino acids within the αV3C stretch are rare in circulating strains

(Supplementary Fig. 4), are not well tolerated in vitro in the absence of an immune response [12] and were not enriched in escape to V3-CD4i bnDs (Fig. 5B).Two strains, Q769_h5 and ZEnv16_1202_7, that remained resistant to optimized bnD.9 indeed have deletions in V3 (Extended Data Fig. 3). These deletions are in principle compatible with helix formation but would be expected to twist the helix interface away of the DARPin and thereby blocking access. A propensity of V3 to form amphipathic helixes has been reported in NMR studies with helices stretching from residues 320-328 [13] and 324-330 [14] and in complex with CCR5 [15], the V3-crown DARPin bnD.2 [5] and the vaccine-induced rabbit antibody 10A37 [16]. While these previously described V3 helixes are considerably shorter than αV3C, they highlight the domain's conserved predisposition to adopt an α-helical structure. Taken together, these lines of evidence suggest a functional significance of the αV3C helix that warrants further investigation to decipher its sensitivity to broad neutralization. Most notably, the similarities of the N-terminal helix turns of αV3C to the described CCR5-bound V3 conformation are striking and suggest a possible involvement of the αV3C region in accessing the co-receptor pocket. Neutralizing antibodies targeting the V3 C-terminal half have not been described in HIV-1 infection. The V3 response is dominated by non-neutralizing N-terminal half and GPG-specific antibodies [17-20]. These antibodies will strongly compete with C-terminal half Abs that bind to αV3C. Although the αV3C region likely requires CD4 triggering for formation and is presumably transiently exposed during entry, our data indicate that antibody access to αV3C is possible (Extended Data Fig. 8). Therefore, further investigation of whether αV3C-directed antibodies develop *in vivo* or can be induced by vaccination is highly warranted. This may provide opportunities to create αV3C scaffold immunogens to elicit novel bnAbs that match the properties of V3-CD4i bnDs. Moreover, even though we observe binding mostly on open Env and post-attachment, spontaneous opening of Env and thus also sampling of αV3C pre-attachment may occur [21-23].

The relatively low potency paired with high breadth of αV3C directed bnDs is in line with their limited opportunity for access, and is reminiscent to what has been observed for post-attachment neutralization active antibodies [24]. However, if considered for in vivo application, the present bnDs may be engineered to high potency taking advantage of the versatile DARPin system, by improving affinity and gaining avidity through multimerization [25].

We hypothesize that αV3C directed antibodies, should they indeed evolve in vivo or be inducible by vaccination, will share the features of post-attachment bnAbs and bnDs and be broad but not potent. Given that this type of inhibitor provides an additional layer of viral control by targeting the cell-bound virion, its activity should nevertheless be exploited for prevention, as it may provide another piece of the puzzle on the path to sterilizing immunity through an HIV vaccine.

Collectively, the definition of the αV3C helix illustrates the high conformational diversity of the Env trimer, where flexible subdomains adopt different conformations within the main open and closed states, providing conserved surfaces for recognition.

3) Supplementary Tables

Table S1: Virus panels

Subtype	Tier	Virus	5-virus panel	13-virus panel	42-virus panel	Genbank entry code
AE	2	ZEnv32_0111_5			x	KU600816
B	2	RHPA4259 clone 7		x	x	AY835447
B	2	ZEnv16_1202_7			x	KU600818
G	2	NAB13pre_cl_9			x	EU023937
B	1B	BaL_26			x	DQ318211
B	2	QH0692 clone 42			x	AY835439
A	2	MG505_W0M_ENV_A2 A1			x	DQ208449
G	2	X2088		x	x	EU885764
B	2	REJO4541 clone 67		x	x	AY835449
A	2	Q769_h5		x	x	AF407159
AE	2	C1080_c03	x	x	x	JN944660
B	2	ZEnv07_0504_15			x	KU600814
B	2	NAB5pre_cl_1			x	EU023923
AG	2	CRF02_cl_250			x	EU513189
C	2	HIV-001428-2			x	EF117266
B	2	TRO clone 11			x	AY835445
C	2	DU422		x	x	AY043175
B	2	JR-CSF			x	AY669726
C	1B	HIV_25925_2			x	EF117273
C	2	ZM106F.PB9			x	AY424163
C	2	53M12		x	x	AY423984
B	3	PVO clone 4			x	AY835444
A	2	KER2018.11			x	AY736810
C	2	ZM214M.PL15			x	DQ3885
BC	1B	CNE40			x	HM215414
B	2	ZEnv91_0505_12			x	KU600815
AE	2	CNE5			x	HM215415
G	2	CRF02_cl_252			x	EU513190
C	2	ZM249M.PL1			x	DQ388514
A1D	unclassified	0815_v3_c3		x	x	HM215260
B	2	NAB9pre_cl_106			x	EU023928
C	2	CAP88.6m.c10			x	KU198436
B	2	JR-FL	x	x	x	AY669728
C	2	ZM233M.PB6		x	x	DQ388517
C	2	CAP45_2_00_G3	x	x	x	DQ435682
AE	2	ZEnv92_1008_8			x	KU600817
A	2	Q842_d12_PNS70d A1		x	x	AF407160
C	2	Du156.12			x	DQ411852
A	2	BG505_W6M_ENV_A5_T332N	x	x	x	DQ208456
B	2	WITO4160 clone 33	x		x	AY835451
AE	2	CNE59			x	HM215422
A	1B	Q23_17			x	AF004885

Table S1: Virus panels

Table S2: Neutralization breadth and potency of V3 directed DARPins and antibodies

		V3-CD4i-bnDs										V3-crown bnDs			
Subtype	strain	bnD.8	bnD.9	bnD.10	bnD.11	bnD.12	bnD.13	bnD.14	bnD.15	bnD.9_trc	bnD.9_opt	bnD.4	bnD.1	bnD.2	bnD.3
A	08150303_ACD	68	40	863	402	62	25	9	49	9	30	56	56	13	141
A	53M12	182	140	>10000	3856	55	112	54	138	59	82	7012	>10000	298	>10000
A	BaL_26	100	407	7685	1974	22	390	100	443	83	9	100	2408	324	507
A	BG505_W6M_T332N	187	312	3694	2059	304	340	293	353	147	156	58	94	680	382
A	C1080_c03	>10000	9831	981	5151	224	9483	334	7130	1191	30	153	134	295	>10000
A	CAP45_2_00_G3	58	213	>10000	2138	37	231	46	208	47	17	10	40	108	121
ACD	CAP88.6m.c10	184	2886	581	6400	98	2886	12	2448	75	9	10	15	9	34
AE	CNE40	758	1068	>10000	>10000	37	1068	327	966	21	171	10	8213	499	1199
AE	CNE5	>10000	>10000	1707	6680	>10000	>10000	2555	>10000	1562	153	15	312	32	318
AE	CNE59	4277	2826	3638	2669	2424	372	180	194	242	131	61	2	41	417
AE	CRF02_cl_250	36	1049	>10000	>10000	19	1461	106	835	196	9	107	>10000	132	8038
AE	CRF02_cl_252	>10000	>10000	>10000	>10000	>10000	>10000	>10000	>10000	3441	1729	136	>10000	>10000	>10000
AG	Du156.12	>10000	>10000	>10000	4537	>10000	>10000	>10000	>10000	>10000	1062	116	>10000	>10000	1297
B	DU422	>10000	>10000	>10000	>10000	>10000	>10000	>10000	>10000	9	2310	293	>10000	5605	>10000
B	HIV_25925_2	220	2520	3832	2312	88	2520	166	1890	80	94	52	2541	26	1438
B	HIV-001428-2_C	>10000	>10000	>10000	>10000	2017	>10000	>10000	>10000	322	276	57	>10000	305	3550
B	JR-CSF	112	648	5548	1588	372	843	28	211	60	70	184	591	406	397
B	JR-FL	440	2259	>10000	>10000	286	2408	150	2210	294	98	278	2914	1072	1467
B	KER2018.11	115	3887	1512	3377	1564	934	35	314	45	20	395	12	8	346
B	MG505_W0M_ENV_A2 A1	45	77	1556	819	20	72	72	108	>10000	51	13	25	62	137
B	NAB13pre_cl_9	76	118	7884	264	1257	106	136	12	350	30	76	>10000	3219	>10000
B	NAB5pre_cl_1	241	653	2697	3019	189	511	161	310	>10000	3949	29	8534	150	2994
B	NAB9pre_cl_106	>10000	>10000	>10000	>10000	>10000	>10000	>10000	>10000	117	134	366	4113	75	7321
B	PVO clone 4	316	1757	1874	2738	115	2447	119	384	116	60	109	94	59	485
B	Q23_17	84	290	6492	3368	151	201	82	231	328	36	27	2	314	363
B	Q769_h5	>10000	>10000	6283	>10000	>10000	>10000	>10000	>10000	>10000	>10000	100	>10000	332	>10000
B	Q842_d12_PNS70d A1	38	75	1612	1361	112	74	47	86	25	29	13	234	106	564
C	QH0692 clone 42	52	100	>10000	1759	28	76	252	122	237	15	44	745	99	199
C	REJO4541 clone 67	1075	7984	>10000	9073	3869	>10000	3437	6129	315	445	10	>10000	>10000	726
C	RHPA4259 clone 7	72	70	3148	2774	47	75	176	86	157	40	14	513	27	279
C	TRO clone 11	412	858	5139	3633	407	1551	329	588	185	219	107	>10000	446	3570
C	WITO4160 clone 33	56	344	>10000	961	82	317	67	391	77	23	10	7105	90	251
C	X2088	>10000	>10000	>10000	>10000	>10000	>10000	>10000	>10000	>10000	1365	129	>10000	2489	>10000
C	ZEnv07_0504_15	78	713	4632	1329	118	574	103	665	239	44	51	2523	106	311
C	ZEnv16_1202_7	>10000	>10000	3040	>10000	6885	>10000	>10000	>10000	>10000	>10000	1056	4261	818	1208
C	ZEnv32_0111_5	>10000	>10000	6040	>10000	7956	>10000	>10000	>10000	880	>10000	1629	>10000	479	>10000
C	ZEnv91_0505_12	26	47	3393	683	13	49	167	80	79	14	8982	>10000	>10000	>10000
C	ZEnv92_1008_8	651	4889	2170	1909	>10000	6637	79	6884	395	9	22	2642	547	>10000
C	ZM106F.PB9	7476	>10000	8333	>10000	3569	>10000	>10000	>10000	1108	136	43	>10000	85	1416
G	ZM214M.PL15	>10000	>10000	>10000	>10000	2973	>10000	>10000	>10000	2189	1035	130	>10000	714	1791
G	ZM233M.PB6	21	1485	1805	1120	>10000	1485	122	1391	35	9	146	216	13	44
G	ZM249M.PL1	248	2713	>10000	4590	128	2356	637	2480	97	14	10	>10000	156	1427
	**Breadth**	71%	71%	62%	69%	79%	69%	74%	71%	86%	93%	100%	62%	90%	76%
	**Geomean IC50**	160	616	2956	2160	218	498	129	429	156	78	79	324	168	608
	**Median IC50**	59	683	2687	378	60	384	83	315	147	60	72	591	156	507

		Antibodies																									
Subtype	strain	VRC41.01	SF12	N6	N49_P7	VRC01	PGV04	b12	HJ16	3BNC117	8Anc195	PG9	PG16	PGDM1400	PGT145	VRC26.09	10-1074	2G12	PGT121	PGT128	PGT135	PGT151	2F5	4E10	10E8	b6	447-52D
A	08150303_ACD	>167	>167	0.03	0.05	1.59	1.08	>167	>167	0.07	7.50	7.56	39.05	4.84	1.19	>167	14.59	>167	5.32	83.06	>167	>167	>167	0.06	0.00	>167	>167
A	53M12	>167	2.57	0.22	0.60	3.42	10.23	>167	>167	1.27	>167	0.67	0.30	0.06	12.45	0.00	0.00	>167	0.04	>167	>167	>167	>167	2.49	1.78	>167	>167
A	BaL_26	0.38	0.34	0.05	0.06	0.08	0.44	0.03	>167	0.00	>167	0.49	45.17	1.20	10.63	>167	0.12	1.08	0.05	0.03	0.04	0.06	2.09	2.40	0.09	>167	>167
A	BG505_W6M_T332N	0.47	0.10	0.05	0.12	0.34	0.22	>167	>167	0.04	0.54	0.18	0.20	0.05	0.41	5.32	0.22	1.69	0.16	0.01	0.87	0.02	4.92	5.83	0.33	n.d.	>167
A	C1080_c03	40.05	0.06	0.37	0.37	21.03	14.87	>167	20.63	0.68	3.78	0.02	0.00	0.01	0.25	6.48	>167	>167	>167	0.11	>167	>167	0.83	1.04	0.07	>167	>167
A	CAP45_2_00_G3	79.55	>167	0.10	0.18	57.04	33.71	1.30	47.05	12.05	29.93	0.02	0.00	0.01	0.01	2.53	>167	>167	62.49	>167	>167	0.03	>167	5.82	1.51	>167	>167
ACD	CAP88.6m.c10	0.29	>167	0.18	0.62	6.49	7.88	20.17	0.00	2.01	>167	>167	>167	>167	>167	0.74	0.60	>167	1.01	0.06	>167	0.00	>167	1.02	0.10	>167	>167
AE	CNE40	0.50	22.69	0.05	0.07	0.11	0.32	18.51	n.d.	0.97	>167	>167	0.23	n.d.	39.59	0.48	0.48	>167	0.02	0.02	>167	0.80	84.88	2.55	0.63	>167	>167
AE	CNE5	>167	0.46	0.20	0.34	1.95	2.13	15.82	2.27	3.84	7.00	0.09	0.59	0.01	0.13	0.03	>167	>167	>167	0.22	>167	>167	6.37	4.99	0.65	25.09	0.06
AE	CNE59	55.11	0.30	n.d.	19.18	1.19	1.50	>167	14.55	0.72	1.53	1.14	15.26	0.00	3.65	>167	>167	>167	>167	>167	>167	28.42	0.07	0.16	0.01	>167	27.51
AE	CRF02_cl_250	1.23	>167	0.33	>167	>167	>167	>167	>167	>167	54.01	0.01	0.00	0.01	8.67	0.01	0.00	24.50	0.00	0.00	>167	0.02	11.97	2.90	0.52	n.d.	>167
AE	CRF02_cl_252	0.09	0.25	0.47	>167	14.16	>167	>167	>167	>167	22.45	0.69	0.38	0.80	6.53	>167	0.03	>167	0.04	0.02	0.24	0.03	10.77	17.99	1.69	>167	>167
AG	Du156.12	0.14	>167	0.00	0.05	0.17	0.18	0.27	>167	0.20	3.98	0.73	2.16	0.15	0.86	2.35	0.17	>167	0.02	0.04	>167	0.03	>167	0.57	0.09	>167	>167
B	DU422	0.39	29.93	0.05	79.12	>167	>167	0.31	>167	>167	>167	63.59	>167	12.88	>167	>167	0.81	>167	0.04	0.05	>167	6.56	>167	5.43	0.23	97.09	73.34
B	HIV_25925_2	0.26	>167	0.17	0.12	2.56	1.08	>167	>167	1.31	>167	0.19	0.22	0.03	0.04	2.81	0.09	>167	0.09	0.02	78.61	0.04	>167	0.86	0.42	>167	>167
B	HIV-001428-2_C	0.33	0.10	0.07	0.05	0.07	0.06	>167	n.d.	0.05	>167	0.00	0.04	n.d.	2.02	0.23	0.23	>167	0.18	0.02	0.06	0.12	>167	8.70	4.03	>167	>167
B	JR-CSF	0.12	0.06	0.06	0.08	1.37	0.26	0.23	>167	0.20	>167	0.00	0.00	0.07	0.02	>167	0.08	2.20	0.17	0.01	0.13	0.04	1.82	2.78	0.43	>167	72.47
B	JR-FL	0.81	0.36	0.05	0.04	0.51	0.52	0.06	21.99	0.01	>167	>167	>167	>167	>167	>167	0.33	3.02	0.15	0.06	>167	0.07	3.57	7.52	0.55	>167	15.85
B	KER2018.11	>167	0.03	1.46	1.38	1.99	1.22	53.46	>167	n.d.	n.d.	0.03	0.01	0.03	0.05	>167	n.d.	>167	>167	>167	>167	0.04	19.78	9.81	2.41	n.d.	6.15
B	MG505_W0M_ENV_A2 A1	>167	7.47	0.10	0.12	0.34	0.17	>167	51.95	0.18	>167	0.56	0.09	0.17	6.64	2.58	90.70	>167	0.48	0.09	>167	0.02	1.34	4.97	0.41	>167	>167
B	NAB13pre_cl_9	>167	>167	0.06	0.04	0.28	58.98	>167	>167	4.10	n.d.	2.68	5.00	5.87	0.07	>167	>167	2.50	>167	>167	0.05	0.33	0.41	0.16	0.40	>167	>167
B	NAB5pre_cl_1	0.82	>167	0.97	0.78	13.01	7.22	52.38	26.96	3.29	2.83	>167	>167	6.91	0.43	>167	0.47	>167	0.36	0.02	2.59	>167	4.19	3.74	0.09	n.d.	>167
B	NAB9pre_cl_106	>167	0.00	0.09	0.26	2.81	0.23	>167	39.14	>167	>167	>167	61.97	>167	18.38	>167	0.29	>167	0.25	52.93	>167	30.87	0.70	1.59	0.04	>167	>167
B	PVO clone 4	0.92	0.26	0.44	0.36	2.29	2.46	>167	>167	0.26	3.32	>167	37.58	15.26	2.71	>167	0.90	4.06	0.18	0.04	>167	>167	2.51	49.64	2.99	>167	>167
B	Q23_17	0.09	0.10	0.11	0.12	0.39	0.23	53.99	>167	0.34	n.d.	0.04	0.01	1.34	1.78	48.06	0.05	>167	0.02	0.01	9.63	0.06	2.22	13.91	1.23	n.d.	>167
B	Q769_h5	0.16	>167	0.08	0.50	0.55	0.42	>167	>167	0.18	3.09	0.02	1.13	0.06	0.48	11.55	>167	>167	>167	66.36	>167	>167	20.54	6.66	3.46	n.d.	2.45
B	Q842_d12_PNS70d A1	>167	0.12	0.00	0.05	0.19	0.10	>167	>167	0.05	>167	0.13	0.24	0.36	0.78	>167	>167	>167	0.17	0.09	>167	0.01	23.01	13.97	5.43	>167	30.01
C	QH0692 clone 42	1.46	0.59	0.54	0.65	13.24	3.66	0.99	6.17	1.46	29.15	>167	>167	>167	>167	>167	1.30	4.07	0.94	0.21	>167	0.72	2.28	2.17	0.80	>167	0.04
C	REJO4541 clone 67	>167	0.10	0.06	0.06	0.99	0.16	20.01	23.36	0.36	1.60	0.92	58.04	0.07	0.01	>167	>167	>167	>167	43.82	>167	0.16	0.62	3.24	0.28	>167	>167
C	RHPA4259 clone 7	0.61	0.20	0.05	0.13	0.17	0.08	0.08	0.11	0.09	4.48	>167	23.92	4.41	0.11	>167	0.35	>167	0.04	0.07	>167	0.08	48.64	56.94	1.05	>167	>167
C	TRO clone 11	0.17	0.51	0.17	0.10	1.86	0.61	>167	2.48	0.20	3.95	>167	>167	4.02	0.23	>167	0.11	0.76	0.07	0.02	0.07	>167	>167	0.38	0.03	>167	>167
C	WITO4160 clone 33	0.43	1.73	0.11	0.22	0.29	11.47	63.69	>167	0.21	>167	0.06	0.01	0.04	0.01	>167	2.90	7.87	27.98	25.60	>167	0.05	3.14	0.46	0.55	>167	>167
C	X2088	117.49	>167	0.10	0.10	>167	>167	>167	>167	>167	>167	>167	>167	>167	>167	>167	0.00	>167	0.01	>167	0.02	>167	>167	>167	>167	>167	>167
C	ZEnv07_0504_15	0.59	0.51	0.51	1.84	3.37	1.59	3.68	>167	1.27	>167	46.65	38.20	22.31	0.87	>167	58.60	>167	16.25	12.63	>167	0.02	6.90	19.65	0.75	>167	>167
C	ZEnv16_1202_7	>167	0.04	0.18	0.05	3.90	6.69	>167	1.22	0.16	1.47	0.61	1.67	0.11	0.01	>167	>167	>167	>167	9.98	>167	0.04	1.21	1.69	0.26	>167	>167
C	ZEnv32_0111_5	>167	2.02	0.28	0.30	>167	>167	>167	>167	0.99	5.07	1.40	0.07	>167	>167	>167	>167	>167	>167	>167	>167	>167	0.34	0.52	0.14	87.29	>167
C	ZEnv91_0505_12	>167	0.40	0.13	0.22	3.52	0.98	3.13	21.25	1.09	8.55	35.41	20.88	>167	16.49	>167	>167	>167	>167	>167	>167	62.82	>167	13.19	1.53	>167	1.23
C	ZEnv92_1008_8	>167	0.36	0.48	0.75	3.33	12.77	>167	>167	2.03	27.95	0.16	3.35	0.05	0.07	6.84	>167	>167	>167	0.04	>167	>167	0.77	0.23	0.11	>167	>167
C	ZM106F.PB9	0.09	0.11	0.02	0.13	0.92	0.89	>167	1.49	1.36	>167	2.80	>167	0.12	0.42	0.34	0.03	>167	0.02	0.02	0.26	79.92	>167	7.27	>167	>167	>167
G	ZM214M.PL15	0.73	57.50	0.36	1.95	11.53	2.86	5.12	5.83	1.84	>167	>167	>167	>167	>167	>167	2.74	>167	0.76	6.23	>167	0.07	>167	36.14	2.05	0.11	2.73
G	ZM233M.PB6	>167	>167	0.03	0.52	56.12	>167	>167	>167	38.61	>167	0.15	9.47	6.27	21.23	4.59	>167	>167	>167	>167	2.71	0.08	>167	1.93	0.50	>167	>167
G	ZM249M.PL1	20.23	>167	0.05	0.15	0.66	10.09	26.52	>167	1.18	>167	2.43	6.48	53.81	1.26	1.20	>167	>167	10.69	1.94	>167	0.04	>167	5.14	56.80	n.d.	>167
	**Breadth**	67%	71%	98%	95%	90%	86%	48%	40%	86%	48%	74%	79%	76%	83%	43%	64%	24%	71%	79%	31%	74%	64%	98%	95%	10%	26%
	**Geomean IC50**	0.86	0.35	0.10	0.25	1.49	1.26	3.09	2.90	0.46	6	0.30	0.62	0.30	0.60	1.00	0.24	3.07	0.20	0.20	0.40	0.14	3.04	2.95	0.44	12.35	4.28
	**Median IC50**	0.48	0.32	0.10	0.16	1.72	1.08	4.40	14.55	0.70	4.23	0.49	0.59	0.13	0.78	2.44	0.29	2.76	0.16	0.06	0.24	0.06	2.51	3.24	0.51	56.19	11.00

Table S2: Neutralization breadth and potency of V3 directed DARPins and antibodies IC_{50} summary of DARPin and mAb activity against the 42-virus multi-clade panel (Figure 1). Maximum concentrations of inhibitors probed were 10’000 nM for DARPins and 167 nM (25 µg/mL) for antibodies. If no inhibition >50% was obtained at the highest concentration tested, this is listed as “>10’000 nM” or “>167 nM”, respectively. Data are derived from one to three independent experiments. nd: not determined

Table S3: Neutralization of JRCSF variants

	Antibodies (µg/mL)			DARPins (µM)				
	**17b**	**447-52D**	**PGT128**	**bnD.2**	**bnD.3**	**bnD.4**	**bnD.8**	**bnD.9**
JRCSF WT	2.987	12.570	0.0077	0.4520	0.2254	0.6422	0.0722	2.8990
L125A	0.006	0.006	0.0165	0.1039	0.0254	0.0159	0.0018	0.0313
N156A	0.029	0.006	0.0115	0.0456	0.0161	0.0285	0.0005	0.0818
Y177A	0.019	0.006	0.0222	0.0212	0.0122	0.0160	0.0001	0.0127
N197K	0.129	0.005	0.0153	0.1067	0.0559	0.0725	0.0012	0.2066
S199A	2.630	0.010	0.0221	0.1701	0.0596	0.1167	0.0083	0.4807
T202A	0.069	0.006	0.0109	0.0701	0.0632	0.0550	0.0119	0.2926
N301A	0.169	0.006	0.0961	0.1279	0.0299	0.0274	0.0021	0.1343
T303A	0.088	0.006	0.1072	0.0474	0.0234	0.0278	0.0027	0.2525
K305A	0.018	0.006	0.0140	0.0663	0.0416	0.0321	0.0046	0.0808
I307A	0.067	0.006	0.0889	0.0437	0.0030	0.0113	0.0007	0.0555
I309A	0.029	0.006	0.0104	1.8989	0.2557	0.0289	0.0006	0.0882
Y318A	0.134	0.006	0.0108	5.1047	3.1577	3.5240	0.0046	0.1604
I323A	0.019	0.006	0.0124	0.0395	0.0275	0.0276	0.0031	0.1756
G324A	0.081	0.006	0.0238	0.0266	0.0265	0.0182	0.0025	0.0758
I420A	21.795	0.006	0.0171	0.5511	0.1390	0.6092	0.0197	0.4383
K421A	0.220	0.006	0.0121	0.3461	0.0329	0.0905	0.0008	0.4289
I423A	1.096	0.006	0.0285	0.0653	0.0494	0.0108	0.1871	0.5721
I424A	4.239	0.006	0.0107	0.2196	0.0863	0.3379	0.0169	0.4613
Y435A	6.251	0.006	0.0065	0.1926	0.1539	0.2663	0.0144	0.6848

Table S3: Neutralization of JRCSF variants Neutralization potency of bnD.8, bnD.9 and antibodies against a panel of alanine mutants in JRCSF Env. Maximum concentrations of inhibitors probed were 10,000 nM for DARPins and 167 nM (25 µg/mL) for antibodies. IC50 values shown are means from two to three independent experiments.

Table S4: Inhibitor Source

Name	Epitope	Reference	Source
b12	CD4bs	Barbas III et al. 1992 PNAS. 89(19):9339-43	D. Burton, The Scripps Research Institute, La Jolla, USA
b6	CD4bs	Barbas III et al. 1992 PNAS. 89(19):9339-43	D. Burton, The Scripps Research Institute, La Jolla, USA
3BNC117	CD4bs	Scheid et al. 2011 Science 16;333(6049):1633-7	M. Nussenzweig, The Rockefeller University, New York, USA
PGV04	CD4bs	Wu et al. 2010 Science. 329(5993):856-61	J. Mascola*, Vaccine Research Center, National Institutes of Health, Bethesda, USA
VRC01	CD4bs	Wu et al. 2010 Science. 329(5993):856-61	J. Mascola*, Vaccine Research Center, National Institutes of Health, Bethesda, USA
NIH45-46	CD4bs	Scheid et al. 2011 Science 16;333(6049):1633-7	M. Nussenzweig, The Rockefeller University, New York, USA
CD4-IgG2	Cd4bs	Allaway et al. 1995 AIDS Res Hum Retroviruses	W. Olson*, Progenics Pharmaceuticals Inc
17b	CD4i	Thali et al. 1993 J Virol. 67(7):3978-88	J. Robinson, Tulane University Medical Center, New Orleans, USA
48d	CD4i	Thali et al. 1993 J Virol. 67(7):3978-88	J. Robinson, Tulane University Medical Center, New Orleans, USA
2G12	High Mannose Patch	Trkola et al. 1996 J Virol. 70(2):1100-8	H. Katinger and D. Katinger, Polymun, Vienna, Austria
8ANC195	Interface/Fusionpeptide	Scharf et al. 2014 Cell Rep. 7(3): 785–795.	Own production
35O22	Interface/Fusionpeptide	Huang et al. 2014 Nature. 515(7525):138-42	J. Mascola*, Vaccine Research Center, National Institutes of Health, Bethesda, USA
PGT151	Interface/Fusionpeptide	Falkowska et al. 2014 Immunity 40(5): 657–668.	Own production
10E8	MPER	Huang et al. 2012 Nature. 491(7424):406-12	12 M. Connors*, National Institute of Allergy and Infectious Diseases, NIH, Bethesda, USA
4E10	MPER	Stiegler et al. 2001 AIDS Res Hum Retroviruses. 17(18):1757-65	H. Katinger and D. Katinger, Polymun, Vienna, Austria
2F5	MPER	Buchacher et al. 1994 AIDS Res Hum Retroviruses. 10(4):359-69	H. Katinger and D. Katinger, Polymun, Vienna, Austria
VRC26.09	V2 Glycan	Doria-Rose et al., 2014 Nature 509, 55-62	L.Morris, P. Moore, National Institute for Communicable Diseases, South Africa
VRC26.25	V2 Glycan	Doria-Rose et al., 2014 Nature 509, 55-62	L.Morris, P. Moore, National Institute for Communicable Diseases, South Africa
PGT145	V2 Glycan	Walker et al. 2011 Nature. 477(7365):466-70	D. Burton, The Scripps Research Institute, La Jolla, USA
PG16	V2 Glycan	Walker et al. 2009 Science. 326(5950):285-9	D. Burton, The Scripps Research Institute, La Jolla, USA
PG9	V2 Glycan	Walker et al. 2009 Science. 326(5950):285-9	D. Burton, The Scripps Research Institute, La Jolla, USA
PGT135	V3 High Mannose Patch	Walker et al. 2011 Nature. 477(7365):466-70	D. Burton, The Scripps Research Institute, La Jolla, USA
PGT128	V3 High Mannose Patch	Walker et al. 2011 Nature. 477(7365):466-70	D. Burton, The Scripps Research Institute, La Jolla, USA
10-1074	V3 High Mannose Patch	Mouquet et al. PNAS 2012 109 (47) 19059-19060;	M. Nussenzweig, The Rockefeller University, New York, USA
PGT121	V3 High Mannose Patch	Walker et al. 2011 Nature. 477(7365):466-70	D. Burton, The Scripps Research Institute, La Jolla, USA
447-52D	V3-Crown	Gorny et al. 1992 J Virol. 66(12):7538-42	H. Katinger and D. Katinger, Polymun, Vienna, Austria
1-79	V3-Crown	Scheid et al. 2009 Nature. 458(7238):636-40	M. Nussenzweig, The Rockefeller University, New York, USA

* Through the NIH AIDS Reagent Program, Division of AIDS, NIAID, NIH

4) Supplementary Figures

S1: Flow cytometry and gating strategy

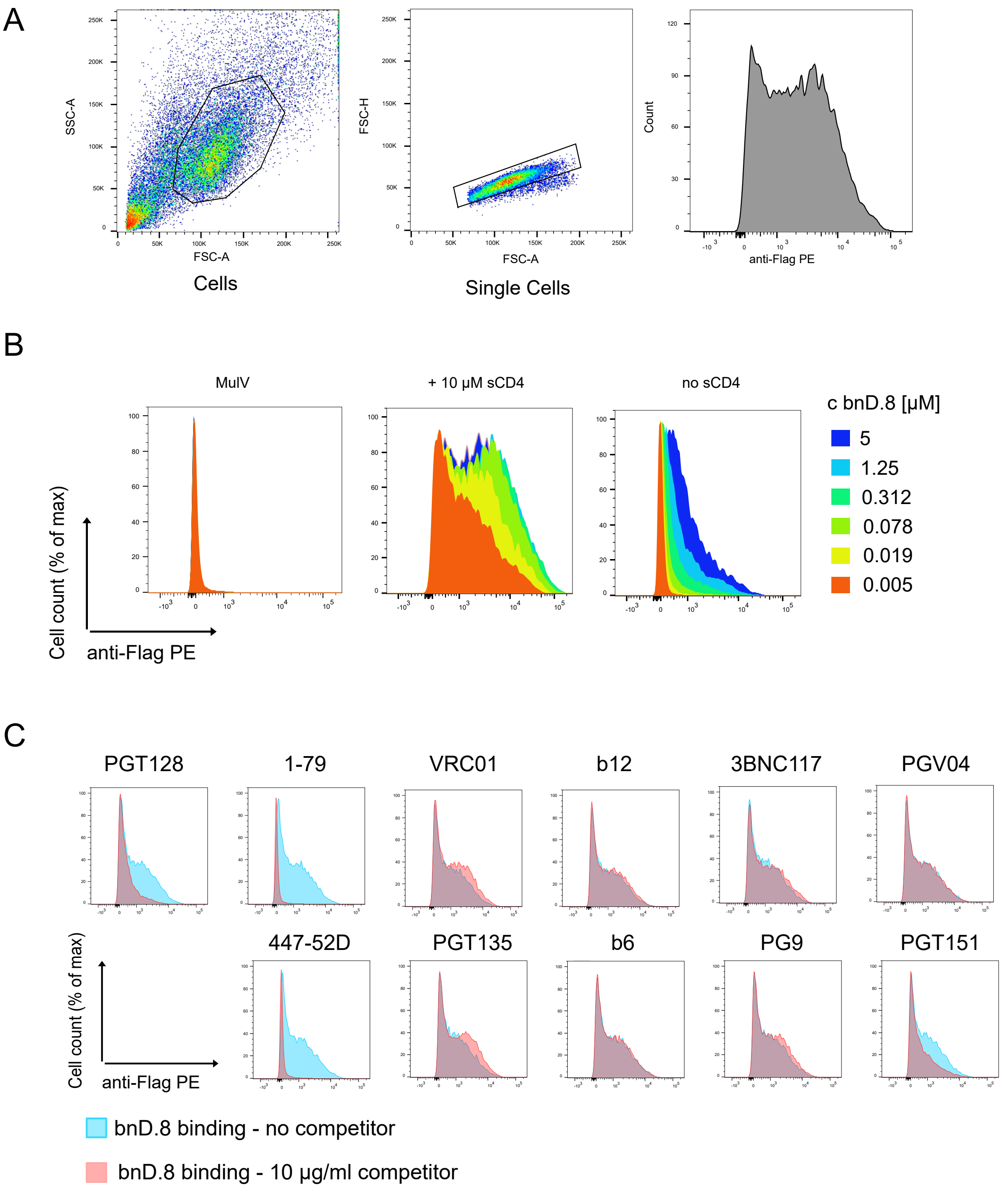

Supplementary Fig. 1. Flow cytometry and gating strategy A) Gating strategy to obtain single cells from the pool of HEK 293 T cells harvested 36h post-transfection with Env-expression plasmid**. B)** bnD.8 binding to SF162 in the presence or absence of 10 µM sCD4. Mulv Env served as negative control. Histograms of normalized fluorescence intensities are shown. Related to Fig. 2C **C)** Binding of 10 µM bnD.8 in the presence or absence of 10 µg/ml competitor antibodies. Histograms of normalized fluorescence intensities are shown. Related to Fig. 2F

S2: Sequence entropy in V3 and the CD4 binding site

V3 residue	Sequence entropy based on 42 virus panel	Sequence entropy based on M env strains	X5	CCR5	17b	bnD.9	10A35	bnD.2	bnD.3	447-52D	268-D
296	0.04	0.01									
297	0.39	0.37									
298	0.04	0.01									
299	0.16	0.09									
300	0.46	0.54									
301	0.09	0.1									
302	0.04	0.1									
303	0.09	0.13									
304	0.09	0.14		x						x	x
305	0.35	0.49		x	x				x	x	x
306	0.09	0.32		x	x				x	x	x
307	0.21	0.39		x	x			x	x	x	x
308	0.62	0.72		x	x		x	x	x	x	x
309	0.21	0.36		x	x	x	x	x	x	x	x
312	0.09	0.08		x		x	x	x	x	x	x
313	0	0.14		x		x	x	x	x	x	x
314	0.04	0.04		x		x	x	x	x	x	x
315	0.34	0.52		x		x	x	x	x	x	x
316	0.42	0.53	x	x		x		x	x		
317	0.13	0.33		x		x	x	x	x	x	x
318	0.12	0.23		x		x	x	x	x	x	
319	0.37	0.44	x	x		x	x	x			
320	0.09	0.3	x	x		x	x	x			
321	0.24	0.4				x	x				
321a	0.52	0.74	x				x				
322	0.04	0.17		x		x	x				
323	0.15	0.26	x	x		x					
324	0	0.05		x		x					
325	0.31	0.28		x							
326	0.09	0.1	x	x	x	x					
327	0.04	0.05	x	x		x					
328	0.37	0.47			x						
329	0.04	0.02									
330	0.25	0.35									
331	0	0.01									

CD4bsresidue	Sequence entropy based on 42 virus panel	Sequence entropy based on M env
102	0.36	0.34
124	0.04	0.05
125	0.04	0.06
126	0.04	0.01
127	0.04	0.04
128	0.04	0.12
194	0.16	0.24
196	0	0.02
257	0.04	0.01
279	0.3	0.4
280	0.09	0.08
281	0.46	0.5
283	0.41	0.45
364	0.27	0.28
365	0	0.02
366	0.04	0.01
367	0.04	0.01
369	0.04	0.02
370	0.26	0.23
424	0.15	0.18
425	0.16	0.31
426	0	0.03
427	0.04	0.06
428	0.44	0.62
429	0.19	0.2
430	0.04	0.05
431	0.47	0.52
454	0.26	0.32
455	0.09	0.12
456	0.04	0.04
457	0.04	0.08
458	0.13	0.17
459	0.88	0.62
460	0.75	0.87
468	0	0.04
470	0.19	0.43
471	0.04	0.02
472	0.04	0.02
473	0.3	0.32
475	0.31	0.32

Supplementary Fig. 2: Sequence entropy in V3 and the CD4 binding site A) Sequence entropy of V3 residues based on the 42-virus panel (Table S1) and all 7590 group M strains listed in the NIH sequence database as of May 2021 are shown. Residues that are recognized by an antibody/DARPin/CCR5 (with non-zero buried surface area) are marked as “x” in the corresponding column. **B)** Sequence entropy of CD4bs epitope residues based on the 42-virus panel and group M strains are shown.

S3: V4-CD4i bnDs are differentially influenced by N-cap framework mutations or removal

A

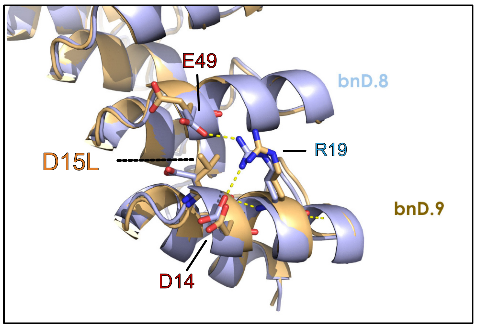

C

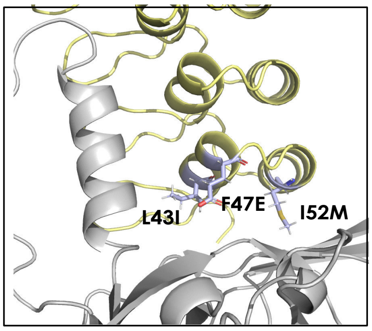

B

IC50 (μM) of bnD.8 and bnD.9 variants against a 15-virus panel

		bnD.9	bnD.9_trc	bnD.9 L15D	bnD.8	bnD.8_trc	bnD.8 D15L
BG505.W6M.C2	A	1.558	0.211	0.121	0.219	0.071	0.111
Q769.h5	A	>10	>10	>10	>10	>10	>10
Q842.d12	A	0.319	0.041	0.052	0.013	0.012	0.010
0815.v3.c3	ACD	0.174	0.012	0.018	0.007	0.002	0.001
C1080.c3	AE	>10	4.977	2.324	>10	0.993	>10
JRFL.JB	B	5.650	0.142	0.385	0.245	0.024	5.351
REJO.67	B	6.734	0.118	2.776	0.815	0.044	>10
RHPA.7	B	0.371	0.092	0.066	0.047	0.007	0.653
CAP45.G3	C	0.919	0.012	0.019	0.024	0.004	0.559
DU422.01	C	>10	>10	7.950	>10	>10	>10
ZM233.6	C	2.421	0.027	0.402	0.008	0.001	0.058
ZM53.12	C	1.242	0.164	0.094	0.083	0.014	0.223
X2088.c9	G	>10	>10	>10	>10	>10	>10
MLV		>10	>10	>10	>10	>10	>10
Median IC50		1.242	0.105	0.121	0.047	0.013	0.167
Sensitive Strains		9	10	11	9	10	8
Resistant Strains		4	3	2	4	3	5
Breadth		69%	77%	85%	69%	77%	62%

D

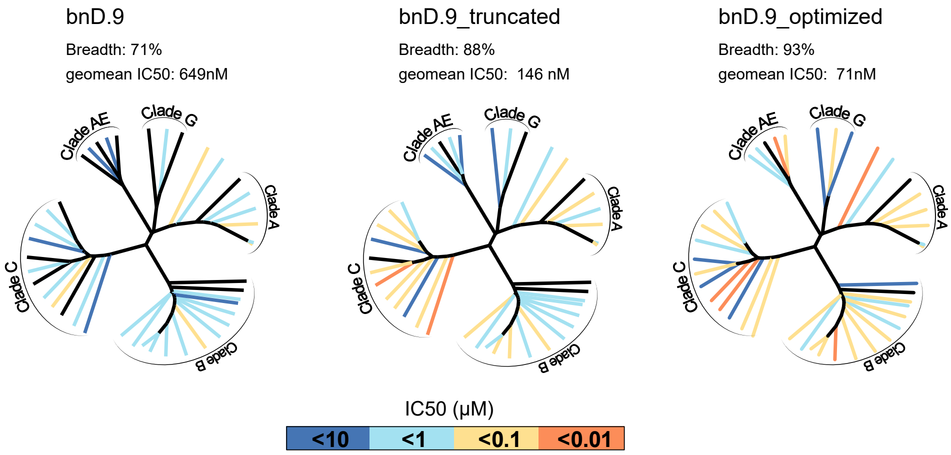

Supplementary Fig. 3: V4-CD4i bnDs are differentially influenced by N-cap framework mutations or removal A) Rearrangement of interactions between the first (N-cap) and second ankyrin repeat observed as a consequence of the bnD.9 backbone mutation D15L. Comparison between bnD.8 (light blue) and bnD.9 (gold) depicts loss of salt bridges to Arg19 by the D15L mutation in bnD.9 **B)**Neutralization capacity of bnD.9 and bnD.8 N-cap variants. Variants of bnD.8 and bnD.9 with the N-cap deleted (bnD.8_trc and bnD.9_trc) as well as bnD.9 L15D and bnD.8 D15L versions were compared to the parental constructs. Median IC50s were calculated over all sensitive strains. **C)** Location of interface mutations introduced into bnD.9_trc to optimize binding to gp120 as defined by Rosetta. Changed residues are shown as sticks and labels indicating the mutation. **D)** Comparison of neutralization capacity of bnD.9 variants on the 42-virus panel. IC50 values of three bnD.9 variants are indicated for 42 virus strains by color code on the phylogenetic trees.

S4: Conservation of V3

	HXB2	A	C	D	E	F	G	H	I	K	L	M	N	P	Q	R	S	T	V	W	Y	gap						
296	C	0	99.88	0	0	0.02	0	0	0	0	0	0	0	0	0	0.04	0	0	0	0	0.06	0	99.9	C	0.06	Y	0.04	R
297	T	1.19	0	0	0.68	0.02	0	0.02	12.2	0.08	0.2	1.02	0	0	0.06	0.06	1.83	79.8	2.83	0	0.04	0	79.8	T	12.17	I	2.83	V
298	R	0	0	0	0	0	0.08	0	0	0.12	0	0	0	0	0	99.8	0	0.02	0	0	0	0	99.8	R	0.12	K	0.08	G
299	P	0.06	0	0	0	0.1	0	0.42	0.06	0	0.94	0	0.1	98	0	0	0	0.5	0.24	0	0.04	0	97.6	P	0.94	L	0.5	T
	-	0	0	0	0	0.02	0.02	0	0	0	0	0	0.1	0	0	0	0	0	0	0	0.02	100	0.06	N	0.02		0.02	
300	N	0.6	0.02	0.54	0	1.31	16.6	1.14	0.06	0.02	0	0	60	0	0.16	0.04	16.4	0.28	0	0	2.47	0	60.4	N	16.55	G	16.37	S
301	N	0	0	0.42	0.3	0	0.04	0.12	0.22	0.82	0	0.02	97	0	0.08	0.22	0.3	0.56	0.08	0	0.14	0.1	96.6	N	0.82	K	0.56	T
302	N	0.02	0.02	0.04	0	0	0.06	0.22	0.2	1.1	0	0	97	0	0	0.18	0.32	0.44	0.04	0	0.46	0	96.9	N	1.1	K	0.46	Y
303	T	0.06	0	0	0.08	0	0.02	0	1.85	1.08	0.06	0.16	0	0	0.14	0.76	0.16	95.1	0.54	0	0	0	95.1	T	1.85	I	1.08	K
304	R	0.08	0	0	0.08	0.02	0.16	0	1.33	0.9	0.24	0.14	0	0	0.12	95.3	1.29	0.14	0.12	0	0	0	95.3	R	1.33	I	1.29	S
305	K	0.08	0	0.04	1.71	0	0.14	0.04	0.48	67.2	0.04	0.08	0.3	0	4.06	12.6	0.14	12.9	0.16	0	0.02	0	67.2	K	12.94	T	12.6	R
306	R	0.06	0	0.24	0.06	0.02	14.3	0.04	0.18	0.32	0	0.02	0.2	0.1	0.14	4.04	80	0.14	0.04	0.02	0.06	0	80	S	14.34	G	4.04	R
	-	0	0	0	0	0	0.04	0.04	0.2	0.02	0	0	0	0	0	0.22	0.12	0.02	0.02	0	0	99	0.22	R	0.2	I	0.12	S
307	I	0.18	0	0	0.1	0.28	0	0.16	71.6	0.04	0.88	3.86	0	0	0	0.08	0.1	3.11	19.4	0.08	0.1	0	71.6	I	19.43	V	3.86	M
308	R	0.54	0	0.02	0	0.02	1.99	31.4	0.12	0.06	0.06	0.04	4.7	6.7	1.15	41.6	4.48	6.59	0.08	0.06	0.48	0	41.6	R	31.38	H	6.71	P
309	I	0	0	0	0	2.05	0.14	0	79.2	0.04	7.85	9.28	0	0	0	0.18	0.04	0.34	0.86	0	0.02	0	79.2	I	9.28	M	7.85	L
310	Q	0.02	0	0	0	0	0.58	0.2	0	0	0	0	0	0	0.02	0.06	0	0	0.02	0	0	99	99.1	-	0.58	G	0.2	H
311	R	0.02	0	0	0	0	0.02	0.02	0.52	0	0.02	0	0	0.1	0	0.12	0	0.04	0.04	0	0	99	99.1	-	0.52	I	0.12	R
312	G	2.77	0	0	0.02	0	96.6	0.02	0.04	0	0	0	0	0	0.06	0.12	0.08	0.16	0.18	0	0	0	96.6	G	2.77	A	0.18	V
313	P	0.26	0	0	0	0.28	0.16	0.02	0.1	0.04	0.98	0.02	0	95	1.25	0.42	0.18	0.16	0.12	1.1	0.02	0	94.9	P	1.25	Q	1.1	W
314	G	0.04	0	0	0.04	0	99.5	0	0	0.04	0.06	0.02	0	0	0	0.14	0	0	0	0.06	0	0.1	99.5	G	0.14	R	0.06	W/L
315	R	0.56	0	0	0.06	0.02	1.53	0.86	0.02	5.54	0.1	0	0	0.1	55.6	32.9	2.45	0.06	0.02	0	0.08	0	55.6	Q	32.92	R	5.54	K
316	A	52.25	0	0	0.08	0.12	0.22	0.02	0.5	0.1	0.22	1.02	0	0.2	0.02	0.26	1.95	29.7	13.1	0.08	0.06	0.1	52.3	A	29.69	T	13.12	V
317	F	0.14	0.02	0	0	82	0.02	0	2.97	0	6.35	0.54	0	0	0.04	0.04	0.18	0	0.82	5.36	1.51	0	82	F	6.35	L	5.36	W
318	V	0.1	0	0.16	0	9.6	0	1.67	0.26	0	0.22	0.06	0	0	0.1	0.14	0.24	0.12	0.42	0.4	86.5	0	86.5	Y	9.6	F	1.67	H
319	T	67.36	0.02	0.02	0.04	0.1	0.98	0.08	0.14	1.57	0.14	0.08	0	0.1	0.48	7.61	0.5	18.6	0.56	0	0.64	0.9	67.4	A	18.64	T	7.61	R
320	I	2.45	0	0.04	0.2	0.02	0.7	0.76	0.64	0.34	0.1	0.8	3	0.5	0.24	1.87	1.04	86.7	0.12	0.04	0.4	0.1	86.7	T	3.01	N	2.45	A
321	G	0.42	0	3.74	2.91	0.02	77.9	0.06	0.02	1.81	0.02	0	5.3	0.2	0.4	1.29	0.66	1.04	0	0	0.08	4.1	77.9	G	5.26	N	3.74	D
321a		7.39	0	45	22.1	0	4.94	0.2	0.08	2.11	0	0.04	2	0	5.6	2.07	1.35	0.68	0.12	0	0.06	6.2	45	D	22.06	E	7.39	A
322	K	0.02	0	0	0.02	0	0	0	92.5	0.1	0.12	0.18	0	0	0.04	0.08	0	0.12	6.45	0	0.02	0.3	92.5	I	6.45	V	0.18	M
323	I	0.08	0	0.08	0.18	0.02	0.18	0	86.7	0.54	0.34	0.22	0.1	0	0.08	0.24	0.06	6.69	4.36	0	0.02	0.1	86.7	I	6.69	T	4.36	V
324	G	0.02	0	0.14	0.12	0	99.2	0	0.02	0.1	0	0	0	0	0.02	0.28	0.04	0.06	0	0	0	0	99.2	G	0.28	R	0.14	D
325	N	0.04	0	80	0.82	0	0.12	0.04	0.1	0.78	0	0	17	0.1	0.12	0.06	0.22	0.04	0.02	0	0.12	0.1	80	D	17.36	N	0.82	E
326	M	0.06	0	0.02	0	0	0.04	0	97.4	0.14	0.1	0.16	0	0.7	0	0.1	0.02	0.7	0.38	0	0	0.2	97.4	I	0.72	P	0.7	T
327	R	0.02	0	0	0	0	0.12	0	0.02	1.31	0	0	0	0	0	98.3	0.04	0.08	0	0.02	0	0	98.3	R	1.31	K	0.12	G
328	Q	0.58	0	0.14	3.21	0	0.02	0.32	0.1	20.8	0.64	0.02	0.5	0.1	68.5	4.58	0.36	0.06	0.04	0.02	0.04	0	68.5	Q	20.75	K	4.58	R
329	A	99.56	0	0.02	0	0	0.02	0	0	0	0	0	0	0	0	0	0.12	0.14	0.08	0	0	0	99.6	A	0.14	T	0.12	S
330	H	0.02	0.02	0.02	0.04	1.63	0	70.1	0	0	0.04	0	0.2	0	0.68	0.2	0.62	0	0.02	0.1	26.2	0	70.1	H	26.2	Y	1.63	F
331	C	0	99.78	0.02	0	0.02	0.02	0	0		0	0	0	0	0.02	0.06	0.04	0	0	0	0.04	0	99.8	C	99.78	R	0.06	X

Supplementary Fig. 4: Conservation of V3 The amino acid frequency was determined for all positions (HxB2 numbering) in V3 across group M based on 5067 Env sequences not containing premature stop codons or frameshifts downloaded from the Los Alamos National Library HIV-1 Env sequence collection on May 23rd, 2021.

4) References

1 Leaver-Fay, A. *et al.* ROSETTA3: an object-oriented software suite for the simulation and design of macromolecules. *Methods Enzymol* **487**, 545-574 (2011). https://doi.org:10.1016/B978-0-12-381270-4.00019-6

2 Leman, J. K. *et al.* Macromolecular modeling and design in Rosetta: recent methods and frameworks. *Nature Methods* **17**, 665-680 (2020). https://doi.org:10.1038/s41592-020-0848-2

3 Rispens, T. *et al.* Dynamics of inter-heavy chain interactions in human immunoglobulin G (IgG) subclasses studied by kinetic Fab arm exchange. *J Biol Chem* **289**, 6098-6109 (2014). https://doi.org:10.1074/jbc.M113.541813

4 Roux, K. H., Strelets, L. & Michaelsen, T. E. Flexibility of human IgG subclasses. *J Immunol* **159**, 3372-3382 (1997).

5 Friedrich, N. *et al.* Distinct conformations of the HIV-1 V3 loop crown are targetable for broad neutralization. *Nature Communications* **12** (2021). https://doi.org:10.1038/s41467-021-27075-0

6 Reh, L. *et al.* Capacity of Broadly Neutralizing Antibodies to Inhibit HIV-1 Cell-Cell Transmission Is Strain- and Epitope-Dependent. *PLoS pathogens* **11**, e1004966 (2015). https://doi.org:10.1371/journal.ppat.1004966

7 Gombos, R. B. *et al.* Inhibitory Effect of Individual or Combinations of Broadly Neutralizing Antibodies and Antiviral Reagents against Cell-Free and Cell-to-Cell HIV-1 Transmission. *J Virol* **89**, 7813-7828 (2015). https://doi.org:10.1128/JVI.00783-15

8 Kim, A. S., Leaman, D. P. & Zwick, M. B. Antibody to gp41 MPER alters functional properties of HIV-1 Env without complete neutralization. *PLoS pathogens* **10**, e1004271 (2014). https://doi.org:10.1371/journal.ppat.1004271

9 Lai, R. P. *et al.* A fusion intermediate gp41 immunogen elicits neutralizing antibodies to HIV-1. *J Biol Chem* **289**, 29912-29926 (2014). https://doi.org:10.1074/jbc.M114.569566

10 Huang, J. *et al.* Broad and potent neutralization of HIV-1 by a gp41-specific human antibody. *Nature* **491**, 406-412 (2012). https://doi.org:10.1038/nature11544

11 Krebs, S. J. *et al.* Longitudinal Analysis Reveals Early Development of Three MPER-Directed Neutralizing Antibody Lineages from an HIV-1-Infected Individual. *Immunity* **50**, 677-+ (2019). https://doi.org:10.1016/j.immuni.2019.02.008

12 Haddox, H. K., Dingens, A. S. & Bloom, J. D. Experimental Estimation of the Effects of All Amino-Acid Mutations to HIV's Envelope Protein on Viral Replication in Cell Culture. *PLoS pathogens* **12**, e1006114 (2016). https://doi.org:10.1371/journal.ppat.1006114

13 Vranken, W. F., Budesinsky, M., Fant, F., Boulez, K. & Borremans, F. A. M. The complete Consensus V3 loop peptide of the envelope protein gp120 of HIV-1 shows pronounced helical character in solution. *FEBS Letters* **374**, 117-121 (1995). https://doi.org:10.1016/0014-5793(95)01086-t

14 Catasti, P., Fontenot, J. D., Bradbury, E. M. & Gupta, G. Local and Global Structural Properties of the HIV-MN V3 Loop. *Journal of Biological Chemistry* **270**, 2224-2232 (1995). https://doi.org:10.1074/jbc.270.5.2224

15 Shaik, M. M. *et al.* Structural basis of coreceptor recognition by HIV-1 envelope spike. *Nature* **565**, 318-323 (2019). https://doi.org:10.1038/s41586-018-0804-9

16 Pan, R. *et al.* Increased Epitope Complexity Correlated with Antibody Affinity Maturation and a Novel Binding Mode Revealed by Structures of Rabbit Antibodies against the Third Variable Loop (V3) of HIV-1 gp120. *J Virol* **92** (2018). https://doi.org:10.1128/JVI.01894-17

17 Burke, V. *et al.* Structural basis of the cross-reactivity of genetically related human anti-HIV-1 mAbs: implications for design of V3-based immunogens. *Structure* **17**, 1538-1546 (2009). https://doi.org:10.1016/j.str.2009.09.012

18 Stephenson, K. E. *et al.* Quantification of the epitope diversity of HIV-1-specific binding antibodies by peptide microarrays for global HIV-1 vaccine development. *J Immunol Methods* **416**, 105-123 (2015). https://doi.org:10.1016/j.jim.2014.11.006

19 Jiang, X. *et al.* Conserved structural elements in the V3 crown of HIV-1 gp120. *Nature structural & molecular biology* **17**, 955-961 (2010). https://doi.org:10.1038/nsmb.1861

20 Stanfield, R. L., Gorny, M. K., Zolla-Pazner, S. & Wilson, I. A. Crystal Structures of Human Immunodeficiency Virus Type 1 (HIV-1) Neutralizing Antibody 2219 in Complex with Three Different V3 Peptides Reveal a New Binding Mode for HIV-1 Cross-Reactivity. *Journal of Virology* **80**, 6093-6105 (2006). https://doi.org:10.1128/jvi.00205-06

21 Han, Q. *et al.* Difficult-to-neutralize global HIV-1 isolates are neutralized by antibodies targeting open envelope conformations. *Nature Communications* **10** (2019). https://doi.org:10.1038/s41467-019-10899-2

22 Lu, M. *et al.* Associating HIV-1 envelope glycoprotein structures with states on the virus observed by smFRET. *Nature* **568**, 415-419 (2019). https://doi.org:10.1038/s41586-019-1101-y

23 Munro, J. B. *et al.* Conformational dynamics of single HIV-1 envelope trimers on the surface of native virions. *Science (New York, N.Y.)* **346**, 759-763 (2014). https://doi.org:10.1126/science.1254426

24 Ruprecht, C. R. *et al.* MPER-specific antibodies induce gp120 shedding and irreversibly neutralize HIV-1. *J Exp Med* **208**, 439-454 (2011). https://doi.org:10.1084/jem.20101907

25 Walser, M. *et al.* *Highly potent anti-SARS-CoV-2 multi-DARPin therapeutic candidates* (Cold Spring Harbor Laboratory, 2020).
