# Supplementary material for: Trapping the HIV-1 V3 loop in a helical conformation enables broad neutralization
Source: Nat Struct Mol Biol. 2023 Aug 21;30(9):1323–36. doi: 10.1038/s41594-023-01062-z (PMC10497408; doi:10.1038/s41594-023-01062-z)
Supplement: Supplementary file 2 — Reporting Summary [file 41594_2023_1062_MOESM2_ESM.pdf]

nature portfolio

Corresponding author(s): Alexandra Trkola

Last updated by author(s): Jun 1, 2023

Reporting Summary

Nature Portfolio wishes to improve the reproducibility of the work that we publish. This form provides structure for consistency and transparency in reporting. For further information on Nature Portfolio policies, see our Editorial Policies and the Editorial Policy Checklist.

Statistics

For all statistical analyses, confirm that the following items are present in the figure legend, table legend, main text, or Methods section.

n/a	Confirmed	
☐	☒	The exact sample size (*n*) for each experimental group/condition, given as a discrete number and unit of measurement
☐	☒	A statement on whether measurements were taken from distinct samples or whether the same sample was measured repeatedly
☒	☐	The statistical test(s) used AND whether they are one- or two-sided<br>*Only common tests should be described solely by name; describe more complex techniques in the Methods section.*
☒	☐	A description of all covariates tested
☒	☐	A description of any assumptions or corrections, such as tests of normality and adjustment for multiple comparisons
☒	☐	A full description of the statistical parameters including central tendency (e.g. means) or other basic estimates (e.g. regression coefficient) AND variation (e.g. standard deviation) or associated estimates of uncertainty (e.g. confidence intervals)
☒	☐	For null hypothesis testing, the test statistic (e.g. *F*, *t*, *r*) with confidence intervals, effect sizes, degrees of freedom and *P* value noted<br>*Give P values as exact values whenever suitable.*
☒	☐	For Bayesian analysis, information on the choice of priors and Markov chain Monte Carlo settings
☒	☐	For hierarchical and complex designs, identification of the appropriate level for tests and full reporting of outcomes
☒	☐	Estimates of effect sizes (e.g. Cohen's *d*, Pearson's *r*), indicating how they were calculated

Our web collection on statistics for biologists contains articles on many of the points above.

Software and code

Policy information about availability of computer code

Data collection	Glycan modelling was performed using python package Glycosylator 1.0. Subsequent molecular dynamics simulations were carried out using ACEMD 3.0 software as described in the Materials and Methods section. The RosettaDock procedure was used for the protein-protein docking, Rosetta FastDesign for interface optimization. Cryo-EM data were collected using the Leginon software.
Data analysis	All data analyses are detailed in the Materials and Methods section. Binding and neutralization data was analysed using GraphPad PRISM1 10. Flow cytometry data was analyzed using FlowJo 10 software. The software package dms_tools2 (2.6.6.) was used for analysis of deep mutational scanning data. X-ray diffraction data of protein crystals was processed using XDS (version Jan 31, 2020), Aimless (version 0.7.4) and autoproc (version 2.3.13) . Initial phases were obtained by molecular replacement using PHASER (version 2.8.3) . Structure refinement was done using REFMAC5 (version 5.8.0267) and Phenix-Refine Refine (version 1.19_4080), followed by model building in COOT (version 0.9.3). For Cryo-EM data automated and manual model building were iteratively performed using real space refinement in Phenix (version 1.19) 90 and Coot (version 0.9.4) respectively. Local resolution was estimated using ResMap (version 1.1.4).Half maps were provided to Resolve Cryo-EM tool in Phenix to support manual model building. Geometry validation and structure quality assessment were performed using EMRinger and Molprobity both implemented in Phenix 1.19. Map-fitting cross correlation (Fit-in-Map tool) and figure preparation were carried out using PyMOL (version 2.4.2) and UCSF Chimera (version 1.15)<br>Binding interfaces were analyzed with LigPlot+ (v2.2.5). Other figures of structures were prepared with PyMOL version 2.5.3.

For manuscripts utilizing custom algorithms or software that are central to the research but not yet described in published literature, software must be made available to editors and reviewers. We strongly encourage code deposition in a community repository (e.g. GitHub). See the Nature Portfolio guidelines for submitting code & software for further information.

Data

Policy information about availability of data

All manuscripts must include a data availability statement. This statement should provide the following information, where applicable:

- Accession codes, unique identifiers, or web links for publicly available datasets
- A description of any restrictions on data availability
- For clinical datasets or third party data, please ensure that the statement adheres to our policy

The structural data on DARPin:V3 complexes generated in this study (Fig. 4, 5 and Supplementary Fig. 4, 5) have been deposited in the Protein Data Bank (PDB) database under accession codes 7Z7C (bnD.8:V3 (BF520)) and 8AED (bnD.9:V3 (BG505)). The Cryo-EM structure of bnD.9:BG505.SOSIP.664:sCD4 was deposited in the PDB under 7TXD and the Electron Microscopy Data Bank (EMDB) under EMD-26157. Tables 1 and 2 with corresponding data collection and refinement statistics are included in the manuscript. Other publicly available datasets from the PDB used in this study (Figures 4,5 and 7) are accessible under PDB IDs 6MEO (CCR5:gp120:sCD4), 2B4C (X5:gp120), 2QAD (412D:gp120), 5VN3 (17b:BG505.SOSIP.664:sCD4), 5V6L (10A37:V3), 7B4U (bnD.2:V3), 7B4W (bnD.3:V3), 3MLV (447-52D:V3), 3GO1 (268-D:V3) and 5FUU (PGT151:JR-FL EnvΔCT). Source data for all other figures are provided as Supplementary Tables. Additional source data retrieved from Friedrich et al. (Nat. Commun., 2021) can be found online under https://doi.org/10.1038/s41467-021-27075-0. HIV sequences downloaded from the Los Alamos National Laboratory database are accessible at https://hiv.lanl.gov, the Alphafold model for CD4 can be accessed under https://alphafold.ebi.ac.uk/entry/P01730.

Human research participants

Policy information about studies involving human research participants and Sex and Gender in Research.

Reporting on sex and gender	N/A
Population characteristics	N/A
Recruitment	N/A
Ethics oversight	N/A

Note that full information on the approval of the study protocol must also be provided in the manuscript.

Field-specific reporting

Please select the one below that is the best fit for your research. If you are not sure, read the appropriate sections before making your selection.

☒ Life sciences ☐ Behavioural & social sciences ☐ Ecological, evolutionary & environmental sciences

For a reference copy of the document with all sections, see nature.com/documents/nr-reporting-summary-flat.pdf

Life sciences study design

All studies must disclose on these points even when the disclosure is negative.

Sample size	No sample size calculations were conducted as the study is fully exploratory. Random 192 DARPins were cloned form the initial library and DARPins with interesting selected for further characterization.
Data exclusions	No data was excluded.
Replication	For all experiments shown, all attempts at replication with optimized parameters as detailed in the materials and methods section were successful: Pseudovirus inhibition assays (Figure 1C: n=2-3, 3A: n=2-4, 5E: n=2, 6D, E ,F: n=2, S8B: n=2), DARPin binding ELISA (Figure 2B n=2-3), competition binding (Figure 2D, E and F). Cell-surface binding experiments using flow-cytometry (Figure 2C), representative data from one of two similarly conducted experiments are shown. Deep mutational scanning data (Figure 5,6 and Extended Data Figure 9) was generated from two independently selected and neutralized libraries. PBMC culture escape was performed once (Figure 6D and Extended Data Figure 10), results were validated by pseudovirus inhibition assays (Figure 6E).
Randomization	No randomization was performed.The aim of our study was to define broadly neutralizing DARPin inhibitors and their epitope. Therefore solely DARPins of interest were selected for follow-up.
Blinding	No blinding was performed as we did not conduct size-effect comparisons but focused on investigating individual inhibitors. There was no control group but where applicable control inhibitors were added.

Reporting for specific materials, systems and methods

We require information from authors about some types of materials, experimental systems and methods used in many studies. Here, indicate whether each material, system or method listed is relevant to your study. If you are not sure if a list item applies to your research, read the appropriate section before selecting a response.

Materials & experimental systems

n/a	Involved in the study
☐	☒ Antibodies
☐	☒ Eukaryotic cell lines
☒	☐ Palaeontology and archaeology
☒	☐ Animals and other organisms
☒	☐ Clinical data
☒	☐ Dual use research of concern

Methods

n/a	Involved in the study
☒	☐ ChIP-seq
☐	☒ Flow cytometry
☒	☐ MRI-based neuroimaging

Antibodies

Antibodies used	- mouse anti-FLAG® antibody (Sigma Aldrich, clone M2, Cat#F1804 and Cat#F3165);<br>- polyclonal alkaline phosphatase-conjugated polyclonal goat anti-mouse IgG (whole molecule) secondary antibody (Sigma Aldrich, Cat#A3562);<br>- PE-conjugated anti-FLAG® L5 antibody (Biolegends, San Diego, USA, Cat#637309)<br>- 6x-His Tag Monoclonal Antibody (clone HIS.H8; Invitrogen, Cat# MA1-21315-1MG)<br>- polyclonal anti-human IgG (Fc specific) Alkaline Phosphatase conjugated antibody produced in goat (Sigma-Aldrich Cat#I2136)<br>- References and sources for all HIV-1 Envelope protein specific antibodies used in this study are detailed in Supplementary Table 6.
Validation	Mouse anti-FLAG® IgG1 antibody (Sigma Aldrich, clone M2, Cat#F1804 and Cat#F3165) was validated by the company for ELISA, Immunblotting, Immunprecipitation, Immunohistochemistry, Immuncytochemistry, and Immunfluorescence-Assays and has been optimized for detection of FLAG-tagged proteins in mammalian, plant and bacterial expression systems. The M2 antibody is not Calcium dependent (unlike clone M1) and is able to recognize the FLAG-tag at the N-terminus, C-terminus and at internal sites.<br>All HIV-1 Envelope protein specific antibodies were validated in the references provided in Supplementary Table 6 and their specificity was verified by the use of appropriate background controls in the current study.

Eukaryotic cell lines

Policy information about cell lines and Sex and Gender in Research

Cell line source(s)	HEK 293-T cells were obtained from the American Type Culture Collection and TZM-bl cells through the NIH AIDS Reagent Program. HEK 293T FreestyleTM suspension (293F and Expi293F) cells were purchased from Thermo Fisher. A3.01 cells (A3.01-CCR5) were generated using retroviral transduction as described in Abela et al., Plos Pathogens (2012). Sup.T1 cells were supplied by Jesse Bloom, Fred Hutchinson Cancer Research Center and are described in Boyd et al., J. Virol., (2015), and were originally obtained from the NIH AIDS reagent program.
Authentication	None of the cell lines used were authenticated again after reception from the specified original source.
Mycoplasma contamination	In the Trkola laboratory, cell lines are routinely tested for mycoplasma contamination. No such contamination was detected in the cells used for the present study.
Commonly misidentified lines<br>(See ICLAC register)	No commonly misidentified cell lines were used in the study.

Flow Cytometry

Plots

Confirm that:

☒ The axis labels state the marker and fluorochrome used (e.g. CD4-FITC).

☒ The axis scales are clearly visible. Include numbers along axes only for bottom left plot of group (a 'group' is an analysis of identical markers).

☒ All plots are contour plots with outliers or pseudocolor plots.

☒ A numerical value for number of cells or percentage (with statistics) is provided.

Methodology

Sample preparation	HEK 293-T cells (obtained from the American Type Culture Collection) were co-transfected with the desired Env-expression plasmid and the pCMV-rev expression helper plasmid in 4:1 ratio. After 36h, cells were incubated with DARPins in the presence or absence of sCD4-183 at the indicated concentrations for 20 minutes at RT. Bound DARPins were detected via the FLAG® tag using PE-conjugated anti-FLAG® L5 antibody (Biolegends, San Diego, USA, Cat#637309).
Instrument	FACSVerse system (BD Biosciences)
Software	FlowJo 10 software (FlowJo LLC, Ashland, USA)
Cell population abundance	No post-sort fractions of live single cells were analyzed.
Gating strategy	Transfected HEK 293T cells were gated by FSC/SSC for single cells (eliminating cell debris), which was analyzed for PE-positive cells (Mulv-Envelope transfected cells served as negative control).

☒ Tick this box to confirm that a figure exemplifying the gating strategy is provided in the Supplementary Information.
